# Large Language Models for Rare Disease Diagnosis at the Undiagnosed Diseases Network

**DOI:** 10.1001/jamanetworkopen.2025.28538

**Published:** 2025-08-22

**Authors:** Cathy Shyr, Thomas A. Cassini, Rory J. Tinker, Kevin W. Byram, Peter J. Embí, Lisa Bastarache, Josh F. Peterson, Hua Xu, Rizwan Hamid

**Affiliations:** 1 Department of Biomedical Informatics, Vanderbilt University Medical Center, Nashville, Tennessee; 2 Department of Pediatrics, Vanderbilt University Medical Center, Nashville, Tennessee; 3 Department of Medicine, Vanderbilt University Medical Center, Nashville, Tennessee; 4 Department of Biomedical Informatics and Data Science, Yale School of Medicine, New Haven, Connecticut

## Abstract

This cohort study assesses whether large language models (LLMs) can identify the final diagnosis, based on available clinical information, for patients referred to the Undiagnosed Diseases Network.

## Introduction

Large language models (LLMs) demonstrated strong diagnostic performance on expert-curated case challenges,^1^ but their ability to assist in rare disease diagnosis is underexplored. Medical conditions of patients referred to the Undiagnosed Diseases Network (UDN) are among the most challenging to diagnose. We assessed whether LLMs can identify the final diagnosis for UDN patients based on available clinical information, which may be incomplete and less structured than expert-curated cases, and compared them with historical clinical review.

## Methods

This cohort study included 90 Vanderbilt University Medical Center (VUMC) UDN cases diagnosed between November 15, 2016, and April 26, 2024 (see eTable 1 in Supplement 1 for structured phenotypes). For LLMs, we selected ChatGPT (Chat Generative Pre-trained Transformer), version 4o (August 6, 2024; Open AI [hereafter, *LLM1*]), because of its predecessor’s diagnostic performance,^1^ and Llama 3.1 8B Instruct (December 18, 2024; Meta [hereafter, *LLM2*]) for its open-source efficiency. We used secure instances of both LLMs to ensure patient privacy. We iteratively developed our prompt using established principles.^2^ For each patient, we prompted the LLMs to generate a differential diagnosis based on their clinical summary (Table), a standardized UDN intake document for diagnostic evaluation (eAppendix in Supplement 1). Written informed consent was obtained. This study was approved by the VUMC institutional review board and followed the STROBE reporting guideline.

**Table.** Prompt and Example of an LLM-Generated Differential^a^

Prompt	LLM-generated differential
Instructions: given the information provided below about a patient with an undiagnosed rare disease, generate a list of the most plausible and specific disease diagnoses. 1. First, generate genetic diagnostic hypotheses based on the patient’s medical history, prior genetic test results (if any), and clinical presentation. Avoid general categories like “mitochondrial disorders” or “endocrine disorders.” Instead, provide specific genetic disease names that could explain the patient’s condition. Output the genetic hypotheses in a numbered list with detailed reasoning and next-step recommendations for each to help confirm or rule it out. Rank the list of hypotheses from most to least likely. 2. After generating the genetic diagnostic hypotheses, provide a separate list of nongenetic diagnostic hypotheses. Consider the clinical presentation and any other nongenetic factors that could be contributing to the patient’s condition. Avoid general categories like “endocrine disorders.” Instead, provide specific nongenetic disease names that could explain the patient’s condition. Output the nongenetic hypotheses in a numbered list with detailed reasoning and next-step recommendations for each to help confirm or rule it out. Rank the list of hypotheses from most to least likely. Patient information: [insert patient’s clinical summary here]^b^	### Genetic diagnostic hypotheses 1. **Frontotemporal dementia (FTD):** - The patient’s personality changes, cognitive decline, and imaging findings of frontal and temporal atrophy could suggest FTD. However, the lack of a clear family history makes this less likely. - **Next steps:** genetic testing for mutations in the *MAPT*, *GRN*, and *C9orf72* genes, which are associated with FTD. 2. **Amyotrophic lateral sclerosis (ALS):** - The family history of ALS and the patient’s neurologic symptoms raise the possibility of familial ALS. However, the patient’s presentation is atypical for ALS. - **Next steps:** genetic testing for ALS-associated genes such as *SOD1*, *TARDBP*, *FUS*, and *C9orf72*. ### Nongenetic diagnostic hypotheses 1. **Chronic traumatic encephalopathy (CTE):** - The patient’s history of concussions and cognitive and behavioral changes could suggest CTE, which is associated with repeated head trauma. - **Next steps:** Neuropsychological testing and continued monitoring of symptoms, as there is currently no definitive test for CTE.

Abbreviation: LLM, large language model.

^a^ For both LLMs, we used a temperature setting of 0.

^b^ An example patient summary is provided in the eAppendix in Supplement 1.

Our primary objective was to assess the LLMs’ diagnostic performance and compare it with historical diagnoses made through clinical review by UDN physicians. The primary end point was the proportion of cases in which the LLM-generated differential included the exact final diagnosis (ie, score of 5 on the Bond scale,^3^ a 5-point system on accuracy and helpfulness). Secondary objectives included assessing LLM helpfulness and resource efficiency. A secondary end point was the proportion of helpful differentials, defined as the LLM differential including either the exact (score = 5) or a closely related diagnosis (score = 4) in which the condition was identified correctly but not the specific genetic subtype (eTable 2 in Supplement 1). Other secondary end points were time and cost required to generate differentials.

LLM-generated differentials were randomized and independently scored by 2 blinded physicians (T.A.C. and R.J.T.; a third [K.W.B.] resolved disagreements). We used Cohen κ to assess interrater agreement. With 90 cases and an approximately 5% clinical review diagnostic rate, we hypothesized an LLM diagnostic rate of 10%. Using precision analysis based on the Wilson 95% CI, the half-width of the CI was 6.3%. We used a 2-sided, 1-sample test of proportions at the *P* < .05 level to compare LLMs and clinical reviews. Statistical analyses were performed using R, version 4.4.2.

## Results

Among 90 cases (51.8% female, 48.2% male), the median age at symptom onset was 7.6 months (IQR, 1.0-82.3 months), and the median length of diagnostic odyssey (age at evaluation completion minus age at symptom onset) was 7.6 years (IQR, 4.0-11.9 years). LLM1 and LLM2 achieved diagnostic rates of 13.3% (95% CI, 7.8%-21.9%) and 10.0% (95% CI, 5.4%-17.9%), respectively, with only LLM1 showing a statistically significantly higher rate than 5.6% from clinical review (*P* = .001) (Figure).^3^ LLM1 and LLM2 provided helpful differentials for 23.3% (95% CI, 15.3%-33.7%) and 16.7% (95% CI, 9.9%-26.3%) of cases, respectively. Estimated cost and processing time were $0.03 and 5 seconds per case, respectively, for LLM1 and $0 and 120 seconds, respectively, for LLM2. Interrater agreement for both models was 88% or higher.

**Figure.**
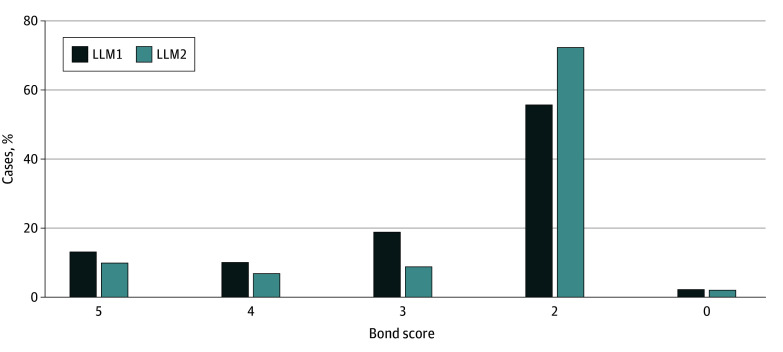
Large Language Model (LLM) Differential Diagnostic Performance Scoring system by Bond et al^3^: 5 = the final diagnosis was suggested in the differential; 4 = the suggestions included something very close to the final diagnosis, but not exact (specifically, this is defined as correctly identifying the condition but not the specific genetic subtype); 3 = the suggestions included something closely related to the final diagnosis that might have been helpful; 2 = the suggestions included something related to the final diagnosis, but unlikely to be helpful; 0 = no suggestion was close to the final diagnosis. A score of 1 is not part of this scoring system. LLM1 indicates ChatGPT (Chat Generative Pre-trained Transformer), version 4o (August 6, 2024; Open AI); LLM2, Llama 3.1 8B Instruct (December 18, 2024; Meta).

## Discussion

LLM1 identified the final diagnosis in 13.3% of cases and provided a helpful differential in 23.3%. Differential generation was resource efficient. These findings suggest that LLM1 can assist clinicians by generating an initial differential and expediting downstream workup. This is the first study to assess LLM diagnostic performance at the UDN. It complements prior studies^1,4,5,6^ by providing insight into the clinical applicability of LLMs. Study limitations include using clinical summaries rather than full workups; the subjectivity of the outcome measure, which was mitigated by standardized scoring; and the retrospective design. Prospective studies are needed to further assess clinical impact.

## Notes

Supplement 1. **eTable 1.** Diagnoses and Structured Phenotypes (i.e., Human Phenotype Ontology (HPO) Terms) of the 90 Cases **eAppendix.** Example Clinical Summary **eTable 2.** Representative Examples of the Bond Scoring Method for Large Language Model (LLM)-Generated Differential Diagnoses

Supplement 2. **Nonauthor Collaborators.** Undiagnosed Diseases Network Members

Supplement 3.
Data Sharing Statement
