# Supplementary material for: Large Language Models for Rare Disease Diagnosis at the Undiagnosed Diseases Network
Source: JAMA Netw Open. 2025 Aug 22;8(8):e2528538. doi: 10.1001/jamanetworkopen.2025.28538 (PMC12374213; doi:10.1001/jamanetworkopen.2025.28538)
Supplement: Supplement 1. — eTable 1. Diagnoses and Structured Phenotypes (i.e., Human Phenotype Ontology (HPO) Terms) of the 90 Cases eAppendix. Example Clinical Summary eTable 2. Representative Examples of the Bond Scoring Method for Large Language Model (LLM)-Generated Differential Diagnoses [file jamanetwopen-e2528538-s001.pdf]

Supplementary Online Content

Shyr C, Cassini TA, Tinker RJ, et al; Undiagnosed Diseases Network. Large language models for rare disease diagnosis at the Undiagnosed Diseases Network. *JAMA Netw Open.* 2025;8(8):e2528538. doi:10.1001/jamanetworkopen.2025.28538

eTable 1. Diagnoses and Structured Phenotypes (i.e., Human Phenotype Ontology (HPO) Terms) of the 90 Cases

eAppendix. Example Clinical Summary

eTable 2. Representative Examples of the Bond Scoring Method for Large Language Model (LLM)-Generated Differential Diagnoses

This supplementary material has been provided by the authors to give readers additional information about their work.

© 2025 Shyr C et al. *JAMA Network Open.*

eTable 1. Diagnoses and Structured Phenotypes (i.e., Human Phenotype Ontology (HPO) terms) of the 90 Cases

	Diagnosis	HPO Term IDs	HPO Term Labels
1	SLC6A1-Related Neurodevelopmental Disorder	HP:0000729,HP:0000750,HP:0001249,HP:0001250,HP:0007359	Autistic behavior,Delayed speech and language development,Intellectual disability,Seizure,Focal-onset seizure
2	BONE MINERAL DENSITY QUANTITATIVE TRAIT LOCUS 18; BMND18	HP:0000682,HP:0005174,HP:0002756,HP:0002757,HP:0002076	Abnormal dental enamel morphology,Membranous subvalvular aortic stenosis,Pathologic fracture,Recurrent fractures,Migraine
3	MUSCULAR DYSTROPHY-DYSTROGLYCANOPATHY (LIMB-GIRDLE), TYPE C, 5; MDDGC5	HP:0012432,HP:0012531,HP:0012544,HP:0001382,HP:0000360,HP:0000545,HP:0001962,HP:0002046,HP:0002094,HP:0002141,HP:0003115,HP:0003201,HP:0003236,HP:0003401,HP:0005307,HP:0007425,HP:0008305,HP:0002321,HP:0002354,HP:0002650,HP:0001166	Chronic fatigue,Pain,Elevated circulating aldolase concentration,Joint hypermobility,Tinnitus,Myopia,Palpitations,Heat intolerance,Dyspnea,Gait imbalance,Abnormal EKG,Rhabdomyolysis,Elevated circulating creatine kinase concentration,Paresthesia,Postural hypotension with compensatory tachycardia,Hyperextensible skin of face,Exercise-induced myoglobinuria,Vertigo,Memory impairment,Scoliosis,Arachnodactyly
4	Nizon-Isidor syndrome	HP:0001263,HP:0001290,HP:0001397,HP:0001513,HP:0007572,HP:0007730,HP:0008751,HP:0100553,HP:0001974,HP:0002015,HP:0000125,HP:0002384,HP:0002870,HP:0002910,HP:0000565,HP:0001000,HP:0032388	Global developmental delay,Generalized hypotonia,Hepatic steatosis,Obesity,Hyperpigmented streaks,Iris hypopigmentation,Laryngeal cleft,Hemihypertrophy of lower limb,Leukocytosis,Dysphagia,Pelvic kidney,Focal impaired awareness seizure,Obstructive sleep apnea,Elevated circulating hepatic transaminase concentration,Esotropia,Abnormality of skin pigmentation,Periventricular nodular heterotopia
5	Diploid-Triploid Mosaicism	HP:0001263,HP:0001290,HP:0001397,HP:0001513,HP:0007572,HP:0007730,HP:0008751,HP:0100553,HP:0001974,HP:0002015,HP:0000125,HP:0002384,HP:0002870,HP:0002910,HP:0	Global developmental delay,Generalized hypotonia,Hepatic steatosis,Obesity,Hyperpigmented streaks,Iris hypopigmentation,Laryngeal cleft,Hemihypertrophy of lower limb,Leukocytosis,Dysphagia,Pelvic kidney,Focal impaired awareness seizure,Obstructive sleep apnea,Elevated circulating hepatic

© 2025 Shyr C et al. *JAMA Network Open.*

		000565,HP:0001000,HP:0032388	transaminase concentration,Esotropia,Abnormality of skin pigmentation,Periventricular nodular heterotopia
6	KABUKI SYNDROME 1; KABUK1	HP:0000218,HP:0000280,HP:0000324,HP:0000407,HP:0000465,HP:0000545,HP:0002777,HP:0002786,HP:0009777,HP:0011471,HP:0001137,HP:0011848,HP:0001257,HP:0001263,HP:0001290,HP:0001357,HP:0001488,HP:0001508,HP:0002943,HP:0003887,HP:0005612,HP:0005856	High palate,Coarse facial features,Facial asymmetry,Sensorineural hearing impairment,Webbed neck,Myopia,Tracheal stenosis,Tracheobronchomalacia,Absent thumb,Gastrostomy tube feeding in infancy,Alternating esotropia,Abdominal colic,Spasticity,Global developmental delay,Generalized hypotonia,Plagiocephaly,Bilateral ptosis,Failure to thrive,Thoracic scoliosis,Abnormal humeral head morphology,Arthrogryposis-like hand anomaly,Ulnar radial head dislocation
7	muscular dystrophy rigid spine and myopathy, congenital with fiber type disproportion	HP:0002942,HP:0002984,HP:0003049,HP:0003198,HP:0003273,HP:0003307,HP:0003458,HP:0005085,HP:0006394,HP:0002058,HP:0002091,HP:0000218,HP:0000220,HP:0000582,HP:0002204,HP:0002578,HP:0002650,HP:0002870,HP:0010290,HP:0011304,HP:0200096,HP:0001265	Thoracic kyphosis,Hypoplasia of the radius,Ulnar deviation of the wrist,Myopathy,Hip contracture,Hyperlordosis,EMG: myopathic abnormalities,Limited knee flexion/extension,Limited pronation/supination of forearm,Myopathic facies,Restrictive ventilatory defect,High palate,Velopharyngeal insufficiency,Upslanted palpebral fissure,Pulmonary embolism,Gastroparesis,Scoliosis,Obstructive sleep apnea,Short hard palate,Broad thumb,Triangular-shaped open mouth,Hyporeflexia
8	NUS1 DEHYDRODOLICHYL DIPHOSPHATE SYNTHASE SUBUNIT; NUS1	HP:0012391,HP:0001251,HP:0001260,HP:0010845,HP:0011471,HP:0000964,HP:0001250,HP:0002317,HP:0002353,HP:0002376,HP:0002522,HP:0000011,HP:0000054,HP:0000316,HP:0000369,HP:0000483,HP:0000540,HP:0002982,HP:0003186,HP:0003324,HP:0003657,HP:0001662,HP:0001763	Hyporeflexia of upper limbs,Ataxia,Dysarthria,EEG with generalized slow activity,Gastrostomy tube feeding in infancy,Eczematoid dermatitis,Seizure,Unsteady gait,EEG abnormality,Developmental regression,Areflexia of lower limbs,Neurogenic bladder,Micropenis,Hypertelorism,Low-set ears,Astigmatism,Hypermetropia,Tibial bowing,Inverted nipples,Generalized muscle weakness,Vascular granular osmiophilic material deposition,Bradycardia,Pes planus

© 2025 Shyr C et al. *JAMA Network Open.*

9	HUNTINGTON DISEASE; HD	HP:0002411,HP:0002650,HP:0001251,HP:0001266,HP:0001332	Myokymia,Scoliosis,Ataxia,Choreoathetosis,Dystonia
10	Spastic paraplegia 35	HP:0000750,HP:0031717,HP:0003487,HP:0006380,HP:0000011,HP:0000358,HP:0002079,HP:0002376,HP:0002518,HP:0002540,HP:0001257,HP:0001270,HP:0001332,HP:0001344,HP:0001371	Delayed speech and language development,Alternating exotropia,Babinski sign,Knee flexion contracture,Neurogenic bladder,Posteriorly rotated ears,Hypoplasia of the corpus callosum,Developmental regression,Abnormal periventricular white matter morphology,Inability to walk,Spasticity,Motor delay,Dystonia,Absent speech,Flexion contracture
11	Congenital disorder of glycosylation, type Ik	HP:0005968,HP:0012412,HP:0001182,HP:0001250,HP:0007359,HP:0008067,HP:0008947,HP:0011344,HP:0100704,HP:0000010,HP:0000011,HP:0000252,HP:0000520,HP:0001649,HP:0002020,HP:0002033,HP:0002069,HP:0001298,HP:0001319,HP:0001344,HP:0001508,HP:0001511,HP:0002357,HP:0002540,HP:0002650,HP:0002714,HP:0002835	Temperature instability,Premature adrenarche,Tapered finger,Seizure,Focal-onset seizure,Abnormally lax or hyperextensible skin,Infantile muscular hypotonia,Severe global developmental delay,Cerebral visual impairment,Recurrent urinary tract infections,Neurogenic bladder,Microcephaly,Proptosis,Tachycardia,Gastroesophageal reflux,Poor suck,Bilateral tonic-clonic seizure,Encephalopathy,Neonatal hypotonia,Absent speech,Failure to thrive,Intrauterine growth retardation,Inability to walk,Scoliosis,Downturned corners of mouth,Aspiration
12	Glutamine related disorder, AD	HP:0002119,HP:0002133,HP:0001263,HP:0001344,HP:0012520,HP:0012762,HP:0030047,HP:0033725,HP:0002384,HP:0006808,HP:0006895,HP:0008936,HP:0500198	Ventriculomegaly,Status epilepticus,Global developmental delay,Absent speech,Dilation of Virchow-Robin spaces,Cerebral white matter atrophy,Abnormal lateral ventricle morphology,Thin corpus callosum,Focal impaired awareness seizure,Cerebral hypomyelination,Lower limb hypertonia,Axial hypotonia,Decreased CSF glutamine concentration
13	15q11.2 Deletion Syndrome	HP:0004322,HP:0006532,HP:0033725,HP:0034295,HP:0001250,HP:0001252,HP:0001263,HP:0000252,HP:0000639,HP:000	Short stature,Recurrent pneumonia,Thin corpus callosum,Reduced cerebral white matter volume,Seizure,Hypotonia,Global developmental

© 2025 Shyr C et al. *JAMA Network Open.*

		1631,HP:0008551,HP:0002650	delay,Microcephaly,Nystagmus,Atrial septal defect,Microtia,Scoliosis
14	Mucopolysaccharidosis type IIIC (Sanfilippo C)	HP:0007018,HP:0008760,HP:0010529,HP:0002059,HP:0004372,HP:0000570,HP:0000716,HP:0000757,HP:0001249,HP:0002186,HP:0002310,HP:0002354,HP:0002362,HP:0002370,HP:0002381,HP:0002870,HP:0001268,HP:0001350	Attention deficit hyperactivity disorder,Violent behavior,Echolalia,Cerebral atrophy,Reduced consciousness,Abnormal saccadic eye movements,Depression,Lack of insight,Intellectual disability,Apraxia,Orofacial dyskinesia,Memory impairment,Shuffling gait,Poor coordination,Aphasia,Obstructive sleep apnea,Mental deterioration,Slurred speech
15	3-methylglutaconic aciduria type 8	HP:0001263,HP:0001266,HP:0001272,HP:0001324,HP:0001337,HP:0001508,HP:0006855,HP:0006895,HP:0008807,HP:0008947,HP:0001250,HP:0002151,HP:0002376,HP:0002827,HP:0002883,HP:0001773,HP:0002063,HP:0002070,HP:0002078,HP:0012751,HP:0030043,HP:0031475,HP:0002944,HP:0005750,HP:0006801,HP:0200049,HP:0200055,HP:0000179,HP:0000369,HP:0000486,HP:0000494	Global developmental delay,Choreoathetosis,Cerebellar atrophy,Muscle weakness,Tremor,Failure to thrive,Cerebellar vermis atrophy,Lower limb hypertonia,Acetabular dysplasia,Infantile muscular hypotonia,Seizure,Increased circulating lactate concentration,Developmental regression,Hip dislocation,Hyperventilation,Short foot,Rigidity,Limb ataxia,Truncal ataxia,Abnormal basal ganglia MRI signal intensity,Hip subluxation,Status epilepticus without prominent motor symptoms,Thoracolumbar scoliosis,Lower-limb joint contracture,Hyperactive deep tendon reflexes,Upper limb hypertonia,Small hand,Thick lower lip vermilion,Low-set ears,Strabismus,Downslanted palpebral fissures
16	SPASTIC PARAPLEGIA 84, AUTOSOMAL RECESSIVE; SPG84	HP:0001347,HP:0001520,HP:0000751,HP:0004482,HP:0007199,HP:0008075,HP:0008969,HP:0002169,HP:0002312,HP:0002317,HP:0002355,HP:0002359,HP:0002370,HP:0002373,HP:0002667,HP:0000012,HP:0000019,HP:0000322,HP:0000403	Hyperreflexia,Large for gestational age,Personality changes,Relative macrocephaly,Progressive spastic paraparesis,Leg muscle stiffness,Clonus,Clumsiness,Unsteady gait,Difficulty walking,Frequent falls,Poor coordination,Febrile seizure (within the age range of 3 months to 6 years),Nephroblastoma,Urinary urgency,Urinary hesitancy,Short philtrum,Recurrent otitis media

© 2025 Shyr C et al. *JAMA Network Open.*

17	Psoriatic Vulgaris	HP:0002315,HP:0002321,HP:0001387,HP:0003323,HP:0007067,HP:0008404,HP:0000958,HP:0000962,HP:0002018,HP:0012432,HP:0012532,HP:0025131,HP:0025493,HP:0033047	Headache,Vertigo,Joint stiffness,Progressive muscle weakness,Distal peripheral sensory neuropathy,Nail dystrophy,Dry skin,Hyperkeratosis,Nausea,Chronic fatigue,Chronic pain,Finger swelling,Palmoplantar erythema,Body ache
18	Plastic bronchitis	HP:0001888,HP:0001962,HP:0002099,HP:0000403,HP:0012263,HP:0006532,HP:0100763,HP:0002835,HP:0000750,HP:0000771	Lymphopenia,Palpitations,Asthma,Recurrent otitis media,Immotile cilia,Recurrent pneumonia,Abnormality of the lymphatic system,Aspiration,Delayed speech and language development,Gynecomastia
19	Early infantile epileptic encephalopathy type 4	HP:0001251,HP:0001263,HP:0001337,HP:0001156,HP:0030084,HP:0002121	Ataxia,Global developmental delay,Tremor,Brachydactyly,Clinodactyly,Generalized non-motor (absence) seizure
20	Autosomal dominant kidney hypomagnesemia (ADKH-RRAGD)	HP:0001644,HP:0000707,HP:0005567,HP:0006530,HP:0006685,HP:0000121	Dilated cardiomyopathy,Abnormality of the nervous system,Renal magnesium wasting,Abnormal pulmonary interstitial morphology,Endocardial fibrosis,Nephrocalcinosis
21	mental retardation, autosomal dominant 31	HP:0002194,HP:0002267,HP:0002307,HP:0002370,HP:0002643,HP:0002020,HP:0001249,HP:0012450,HP:0012683,HP:0030319,HP:0100247,HP:0006863,HP:0008872,HP:0008935,HP:0010862,HP:0100786,HP:0003781,HP:0006460	Delayed gross motor development,Exaggerated startle response,Drooling,Poor coordination,Neonatal respiratory distress,Gastroesophageal reflux,Intellectual disability,Chronic constipation,Pineal cyst,Weakness of facial musculature,Recurrent singultus,Severe expressive language delay,Feeding difficulties in infancy,Generalized neonatal hypotonia,Delayed fine motor development,Hypersomnia,Excessive salivation,Increased laxity of ankles
22	Glycogen Storage disorder	HP:0001988,HP:0002007,HP:0002079,HP:0000639,HP:0004322,HP:0006568,HP:0002645,HP:0000252,HP:0000494,HP:0011800,HP:0001252,HP:0001263,HP:0001290	Recurrent hypoglycemia,Frontal bossing,Hypoplasia of the corpus callosum,Nystagmus,Short stature,Increased hepatic glycogen content,Wormian bones,Microcephaly,Downslanted palpebral fissures,Midface retrusion,Hypotonia,Global developmental delay,Generalized hypotonia

© 2025 Shyr C et al. *JAMA Network Open.*

23	Muscular dystrophy, limb-girdle, type 2Y	HP:0003236,HP:0003323,HP:0003458,HP:0002015,HP:0012544,HP:0045040,HP:0100295,HP:0002515,HP:0002747,HP:0002875,HP:0002910,HP:0000020	Elevated circulating creatine kinase concentration,Progressive muscle weakness,EMG: myopathic abnormalities,Dysphagia,Elevated circulating aldolase concentration,Abnormal circulating lactate dehydrogenase concentration,Muscle fiber atrophy,Waddling gait,Respiratory insufficiency due to muscle weakness,Exertional dyspnea,Elevated circulating hepatic transaminase concentration,Urinary incontinence
24	SPINOCEREBELLAR ATAXIA 28; SCA28	HP:0000217,HP:0000360,HP:0000496,HP:0002321,HP:0002500,HP:0002829,HP:0001324,HP:0001350,HP:0003072,HP:0003077,HP:0004374,HP:0005609,HP:0011850,HP:0012378,HP:0100512,HP:0001677,HP:0002015,HP:0002020,HP:0002036,HP:0002067,HP:0002141,HP:0002150,HP:0000608,HP:0000651,HP:0000822,HP:0000938,HP:0001097	Xerostomia,Tinnitus,Abnormality of eye movement,Vertigo,Abnormal cerebral white matter morphology,Arthralgia,Muscle weakness,Slurred speech,Hypercalcemia,Hyperlipidemia,Hemiplegia/hemiparesis,Gallbladder dysfunction,Parotitis,Fatigue,Decreased circulating vitamin D concentration,Coronary artery atherosclerosis,Dysphagia,Gastroesophageal reflux,Hiatus hernia,Bradykinesia,Gait imbalance,Hypercalciuria,Macular degeneration,Diplopia,Hypertension,Osteopenia,Keratoconjunctivitis sicca
25	Intellectual developmental disorder with dysmorphic facies and behavioral abnormalities	HP:0001263,HP:0001290,HP:0001344,HP:0001513,HP:0000565,HP:0000579,HP:0000540,HP:0004322	Global developmental delay,Generalized hypotonia,Absent speech,Obesity,Esotropia,Nasolacrimal duct obstruction,Hypermetropia,Short stature
26	Rett syndrome, congenital variant	HP:0000565,HP:0000637,HP:0000817,HP:0001249,HP:0001250,HP:0007375,HP:0007655,HP:0011231,HP:0011471,HP:0001263,HP:0001290,HP:0001332,HP:0001344,HP:0001508,HP:0002307,HP:0002376,HP:0002553,HP:0000252,HP:0000286,HP:0000437,HP:0002015,HP:0002072,HP:0002079,HP:0002126	Esotropia,Long palpebral fissure,Reduced eye contact,Intellectual disability,Seizure,Abnormal septum pellucidum morphology,Eversion of lateral third of lower eyelids,Prominent eyelashes,Gastrostomy tube feeding in infancy,Global developmental delay,Generalized hypotonia,Dystonia,Absent speech,Failure to thrive,Drooling,Developmental regression,Highly arched eyebrow,Microcephaly,Epicanthus,De

© 2025 Shyr C et al. *JAMA Network Open.*

			pressed nasal tip,Dysphagia,Chorea,Hypoplasia of the corpus callosum,Polymicrogyria
27	osteopathia striata with cranial sclerosis	HP:0011712,HP:0012430,HP:0040022,HP:0002942,HP:0004322,HP:0004590,HP:0005824,HP:0006156,HP:0001344,HP:0002650,HP:0002779,HP:0008826,HP:0010864,HP:0011386,HP:0011471,HP:0000202,HP:0000238,HP:0000248,HP:0000286,HP:0000337,HP:0000348,HP:0000368,HP:0000437,HP:0000527,HP:0000582,HP:0000648,HP:0000889,HP:0001250,HP:0001643,HP:0001679,HP:0002000,HP:0002015,HP:0002021,HP:0002023,HP:0002119,HP:0002144	Right bundle branch block,Cerebral white matter hypoplasia,Clinodactyly of the 2nd finger,Thoracic kyphosis,Short stature,Hypoplastic sacrum,Clinodactyly of the 2nd toe,Ulnar deviation of thumb,Absent speech,Scoliosis,Tracheomalacia,Dislocation of the femoral head,Intellectual disability, severe,Narrow internal auditory canal,Gastrostomy tube feeding in infancy,Orofacial cleft,Hydrocephalus,Brachycephaly,Epicanthus,Broad forehead,High forehead,Low-set, posteriorly rotated ears,Depressed nasal tip,Long eyelashes,Upslanted palpebral fissure,Optic atrophy,Abnormal clavicle morphology,Seizure,Patent ductus arteriosus,Abnormal aortic morphology,Short columella,Dysphagia,Pyloric stenosis,Anal atresia,Ventriculomegaly,Tethered cord
28	Glomerulocystic kidney disease with hyperuricemia and isosthenuria		
29	Vici syndrome (VICIS)	HP:0003165,HP:0003236,HP:0000011,HP:0000543,HP:0001290,HP:0001332,HP:0002342,HP:0002384,HP:0002505,HP:0002650,HP:0025331,HP:0031717,HP:0000646,HP:0000648,HP:0001007,HP:0002063	Elevated circulating parathyroid hormone level,Elevated circulating creatine kinase concentration,Neurogenic bladder,Optic disc pallor,Generalized hypotonia,Dystonia,Intellectual disability, moderate,Focal impaired awareness seizure,Loss of ambulation,Scoliosis,Upgaze palsy,Alternating exotropia,Amblyopia,Optic atrophy,Hirsutism,Rigidity
30	KCNC2-related disease	HP:0001263,HP:0001298,HP:0001250,HP:0000252,HP:0002079	Global developmental delay,Encephalopathy,Seizure,Microcephaly,Hypoplasia of the corpus callosum
31	Roifman syndrome	HP:0002500,HP:0002572,HP:0002578,H	Abnormal cerebral white matter morphology,Episodic

© 2025 Shyr C et al. *JAMA Network Open.*

		P:0002781,HP:0011968,HP:0012262,HP:0002020,HP:0002069,HP:0002099,HP:0002119,HP:0000252,HP:0000358,HP:0000369,HP:0005263,HP:0001252,HP:0001263,HP:0001508,HP:0001511,HP:0200117	vomiting,Gastroparesis,Upper airway obstruction,Feeding difficulties,Abnormal ciliary motility,Gastroesophageal reflux,Bilateral tonic-clonic seizure,Asthma,Ventriculomegaly,Microcephaly,Posteriorly rotated ears,Low-set ears,Gastritis,Hypotonia,Global developmental delay,Failure to thrive,Intrauterine growth retardation,Recurrent upper and lower respiratory tract infections
32	Ehlers-Danlos Syndrome, Hypermobility Type	HP:0001388,HP:0000131,HP:0000545,HP:0012479,HP:0012712,HP:0031064,HP:0000716,HP:0000939,HP:0000974,HP:0000978,HP:0003124,HP:0005110,HP:0005180,HP:0006460,HP:0002757,HP:0002829,HP:0001634,HP:0001763,HP:0002019,HP:0002076	Uterine leiomyoma,Myopia,Temporomandibular joint crepitus,Mild hearing impairment,Impaired continence,Depression,Osteoporosis,Hyperextensible skin,Bruising susceptibility,Hypercholesterolemia,Atrial fibrillation,Tricuspid regurgitation,Increased laxity of ankles,Recurrent fractures,Arthralgia,Mitral valve prolapse,Pes planus,Constipation,Migraine
33	MRD 26	HP:0000565,HP:0000574,HP:0001238,HP:0000154,HP:0000215,HP:0000316,HP:0000348,HP:0000431,HP:0000455,HP:0000483,HP:0001257,HP:0001263,HP:0001382,HP:0001508,HP:0002996,HP:0004283,HP:0002141,HP:0100543,HP:0008443,HP:0008800,HP:0010501,HP:0002308,HP:0002362	Esotropia,Thick eyebrow,Slender finger,Wide mouth,Thick upper lip vermilion,Hypertelorism,High forehead,Wide nasal bridge,Broad nasal tip,Astigmatism,Spasticity,Global developmental delay,Joint hypermobility,Failure to thrive,Limited elbow movement,Narrow palm,Gait imbalance,Cognitive impairment,Neuropathic spinal arthropathy,Limited hip movement,Limitation of knee mobility,Chiari malformation,Shuffling gait
34	Cortical dysplasia, complex, with other brain malformations 5	HP:0012762,HP:0011471,HP:0002171,HP:0002870,HP:0000252,HP:0002079,HP:0002121,HP:0200136,HP:0001263,HP:0001290,HP:0001336	Cerebral white matter atrophy,Gastrostomy tube feeding in infancy,Gliosis,Obstructive sleep apnea,Microcephaly,Hypoplasia of the corpus callosum,Generalized non-motor (absence) seizure,Oral-pharyngeal dysphagia,Global developmental delay,Generalized hypotonia,Myoclonus
35	Diploid/triploid mosaicism	HP:0001631,HP:0002000,HP:0030148,H	Atrial septal defect,Short columella,Heart murmur,Finger

© 2025 Shyr C et al. *JAMA Network Open.*

		P:0040019,HP:0000028,HP:0000054,HP:0000218,HP:0000286,HP:0000316,HP:0000347,HP:0000368,HP:0000463,HP:0000508,HP:0001263,HP:0001290,HP:0001357,HP:0001388,HP:0001510,HP:0000953,HP:0000973,HP:0007183,HP:0008829,HP:0011081,HP:0004322,HP:0004691,HP:0005280,HP:0006610,HP:0002240	clinodactyly,Cryptorchidism,Micropenis,High palate,Epicanthus,Hypertelorism,Micrognathia,Low-set, posteriorly rotated ears,Anteverted nares,Ptosis,Global developmental delay,Generalized hypotonia,Plagiocephaly,Growth delay,Hyperpigmentation of the skin,Cutis laxa,Focal T2 hyperintense basal ganglia lesion,Delayed femoral head ossification,Incisor macrodontia,Short stature,2-3 toe syndactyly,Depressed nasal bridge,Wide intermamillary distance,Hepatomegaly
36	Van Maldergem syndrome 2	HP:0001631,HP:0002000,HP:0030148,HP:0040019,HP:0000028,HP:0000054,HP:0000218,HP:0000286,HP:0000316,HP:0000347,HP:0000368,HP:0000463,HP:0000508,HP:0001263,HP:0001290,HP:0001357,HP:0001388,HP:0001510,HP:0000953,HP:0000973,HP:0007183,HP:0008829,HP:0011081,HP:0004322,HP:0004691,HP:0005280,HP:0006610,HP:0002240	Atrial septal defect,Short columella,Heart murmur,Finger clinodactyly,Cryptorchidism,Micropenis,High palate,Epicanthus,Hypertelorism,Micrognathia,Low-set, posteriorly rotated ears,Anteverted nares,Ptosis,Global developmental delay,Generalized hypotonia,Plagiocephaly,Growth delay,Hyperpigmentation of the skin,Cutis laxa,Focal T2 hyperintense basal ganglia lesion,Delayed femoral head ossification,Incisor macrodontia,Short stature,2-3 toe syndactyly,Depressed nasal bridge,Wide intermamillary distance,Hepatomegaly
37	EPILEPTIC ENCEPHALOPATHY, EARLY INFANTILE, 28; EIEE28	HP:0007886,HP:0033725,HP:0000252,HP:0000615,HP:0001249,HP:0100704,HP:0001250,HP:0001252,HP:0001344,HP:0002944	Absent extraocular muscles,Thin corpus callosum,Microcephaly,Abnormal pupil morphology,Intellectual disability,Cerebral visual impairment,Seizure,Hypotonia,Absent speech,Thoracolumbar scoliosis
38	Mitochondrial complex I deficiency, nuclear type 21	HP:0100852,HP:0002154,HP:0002355,HP:0002465,HP:0012228,HP:0003348,HP:0003542,HP:0003648,HP:0001251,HP:0001272,HP:0001310,HP:0001328,HP:0008551,HP:0000637,HP:0001249,HP:0002066,	Abnormal fear-induced behavior,Hyperglycinemia,Difficulty walking,Poor speech,Tension-type headache,Hyperalaninemia,Increased serum pyruvate,Lacticaciduria,Ataxia,Cerebellar atrophy,Dysmetria,Specific learning disability,Microtia,Long palpebral fissure,Intellectual disability,Gait

© 2025 Shyr C et al. *JAMA Network Open.*

		HP:0002075,HP:0002080,HP:0002141	ataxia,Dysdiadochokinesis,Intention tremor,Gait imbalance
39	HYPOPHOSPHATASIA, ADULT; HPPA	HP:0003155,HP:0000938,HP:0020110	Elevated circulating alkaline phosphatase concentration,Osteopenia,Bone fracture
40	MENTAL RETARDATION, AUTOSOMAL DOMINANT 26; MRD26	HP:0000565,HP:0000677,HP:0000717,HP:0001249,HP:0001263,HP:0001508,HP:0001513,HP:0000256,HP:0000316,HP:0000483,HP:0000494,HP:0008499,HP:0008551,HP:0010863,HP:0011090,HP:0004467,HP:0001763,HP:0001770,HP:0002020,HP:0002021,HP:0002186,HP:0002474,HP:0002591,HP:0002705,HP:0002857,HP:0002870	Esotropia,Oligodontia,Autism,Intellectual disability,Global developmental delay,Failure to thrive,Obesity,Macrocephaly,Hypertelorism,Astigmatism,Downslanted palpebral fissures,High hypermetropia,Microtia,Receptive language delay,Fused teeth,Preauricular pit,Pes planus,Toe syndactyly,Gastroesophageal reflux,Pyloric stenosis,Apraxia,Expressive language delay,Polyphagia,High, narrow palate,Genu valgum,Obstructive sleep apnea
41	INTELLECTUAL DEVELOPMENTAL DISORDER WITH HYPOTONIA, IMPAIRED SPEECH, AND DYSMORPHIC FACIES; IDDHISD	HP:0012523,HP:0025100,HP:0006610,HP:0001252,HP:0001263,HP:0001531,HP:0000957,HP:0000400,HP:0010296,HP:0010863,HP:0002474,HP:0002572,HP:0001773,HP:0002019	Oral aversion,Abnormal hippocampus morphology,Wide intermamillary distance,Hypotonia,Global developmental delay,Failure to thrive in infancy,Cafe-au-lait spot,Macrotia,Ankyloglossia,Receptive language delay,Expressive language delay,Episodic vomiting,Short foot,Constipation
42	Huntington's Disease	HP:0002376,HP:0001250,HP:0001251,HP:0001321,HP:0001332	Developmental regression,Seizure,Ataxia,Cerebellar hypoplasia,Dystonia
43	NR2F2-associated congenital heart defects	HP:0012332,HP:0012625,HP:0001629,HP:0001631,HP:0001748,HP:0002099,HP:0004209,HP:0002370,HP:0002870,HP:0001256,HP:0001562,HP:0007018,HP:0011471,HP:0000083,HP:0000110,HP:0000252,HP:0000577,HP:0000601,HP:0000846,HP:0000954	Abnormal autonomic nervous system physiology,Stage 3 chronic kidney disease,Ventricular septal defect,Atrial septal defect,Polysplenia,Asthma,Clinodactyly of the 5th finger,Poor coordination,Obstructive sleep apnea,Intellectual disability, mild,Oligohydramnios,Attention deficit hyperactivity disorder,Gastrostomy tube feeding in infancy,Renal insufficiency,Renal dysplasia,Microcephaly,Exotropia,Hypotelorism,Adrenal

© 2025 Shyr C et al. *JAMA Network Open.*

			insufficiency,Single transverse palmar crease
44	NEURODEVELOPMENTAL DISORDER WITH HYPOTONIA, BRAIN ANOMALIES, DISTINCTIVE FACIES, AND ABSENT LANGUAGE; NEDHAFA	HP:0030822,HP:0030880,HP:0000218,HP:0000347,HP:0000400,HP:0000717,HP:0001250,HP:0006956,HP:0007565,HP:0002353,HP:0002518,HP:0001263,HP:0001290,HP:0001344,HP:0001488,HP:0005487,HP:0002079	Hooded upper eyelid,Raynaud phenomenon,High palate,Micrognathia,Macrotia,Autism,Seizure,Lateral ventricle dilatation,Multiple cafe-au-lait spots,EEG abnormality,Abnormal periventricular white matter morphology,Global developmental delay,Generalized hypotonia,Absent speech,Bilateral ptosis,Prominent metopic ridge,Hypoplasia of the corpus callosum
45	Developmental delay, impaired growth, dysmorphic facies, and axonal neuropathy	HP:0008936,HP:0011471,HP:0002376,HP:0005709,HP:0001531,HP:0000319,HP:0002015,HP:0012707,HP:0012747,HP:0012751	Axial hypotonia,Gastrostomy tube feeding in infancy,Developmental regression,2-3 toe cutaneous syndactyly,Failure to thrive in infancy,Smooth philtrum,Dysphagia,Elevated brain lactate level by MRS,Abnormal brainstem MRI signal intensity,Abnormal basal ganglia MRI signal intensity
46	MITCHELL SYNDROME	HP:0000011,HP:0000408,HP:0007141,HP:0009830,HP:0002344,HP:0002756,HP:0001271,HP:0001324,HP:0000939	Neurogenic bladder,Progressive sensorineural hearing impairment,Sensorimotor neuropathy,Peripheral neuropathy,Progressive neurologic deterioration,Pathologic fracture,Polyneuropathy,Muscle weakness,Osteoporosis
47	hypotonia, infantile, with psychomotor retardation and characteristic facies 3	HP:0002263,HP:0002373,HP:0002553,HP:0006970,HP:0008947,HP:0011471,HP:0001763,HP:0002015,HP:0000639,HP:0001250,HP:0001263,HP:0001265,HP:0001344,HP:0001357,HP:0001531,HP:0001601,HP:0012450,HP:0012471,HP:0000218,HP:0000426,HP:0000490	Exaggerated cupid's bow,Febrile seizure (within the age range of 3 months to 6 years),Highly arched eyebrow,Periventricular leukomalacia,Infantile muscular hypotonia,Gastrostomy tube feeding in infancy,Pes planus,Dysphagia,Nystagmus,Seizure,Global developmental delay,Hyporeflexia,Absent speech,Plagiocephaly,Failure to thrive in infancy,Laryngomalacia,Chronic constipation,Thick vermilion border,High palate,Prominent nasal bridge,Deeply set eye
48	Titinopathy	HP:0000023,HP:0002355,HP:0002804,HP:0003307,HP:00012	Inguinal hernia,Difficulty walking,Arthrogryposis multiplex congenita,Hyperlordosis,Hypotonia,Hi

© 2025 Shyr C et al. *JAMA Network Open.*

		52,HP:0001385,HP:0002015,HP:0000938,HP:0008689,HP:0011471	p dysplasia,Dysphagia,Osteopenia,Bilateral cryptorchidism,Gastrostomy tube feeding in infancy
49	GTP cyclohydrolase I deficiency	HP:0006986,HP:0007015,HP:0009062,HP:0002015,HP:0002020,HP:0002061,HP:0000403,HP:0003324,HP:0004373,HP:0001263,HP:0002540,HP:0002870	Upper limb spasticity,Poor gross motor coordination,Infantile axial hypotonia,Dysphagia,Gastroesophageal reflux,Lower limb spasticity,Recurrent otitis media,Generalized muscle weakness,Focal dystonia,Global developmental delay,Inability to walk,Obstructive sleep apnea
50	Lambert Eaton Myasthenic Syndrome	HP:0001271,HP:0001324,HP:0003198,HP:0003394,HP:0003444,HP:0012450	Polyneuropathy,Muscle weakness,Myopathy,Muscle spasm,EMG: chronic denervation signs,Chronic constipation
51	Helsmoortel-Van der Aa Syndrome	HP:0004322,HP:0004482,HP:0004660,HP:0000565,HP:0000598,HP:0000717,HP:0000851,HP:0002778,HP:0001263,HP:0001290,HP:0001510,HP:0001539,HP:0008872,HP:0001655,HP:0001763,HP:0002020,HP:0002025,HP:0000075,HP:0000347,HP:0000494	Short stature,Relative macrocephaly,Hypoplasia of facial musculature,Esotropia,Abnormality of the ear,Autism,Congenital hypothyroidism,Abnormal tracheal morphology,Global developmental delay,Generalized hypotonia,Growth delay,Omphalocele,Feeding difficulties in infancy,Patent foramen ovale,Pes planus,Gastroesophageal reflux,Anal stenosis,Renal duplication,Micrognathia,Downslanted palpebral fissures
52	Mandibulofacial dysostosis, Guion-almeida type	HP:0000252,HP:0000347,HP:0000369,HP:0000413,HP:0000463,HP:0008513,HP:0002230,HP:0004322,HP:0000601,HP:0000750,HP:0001100,HP:0001263,HP:0001629,HP:0001763,HP:0002002	Microcephaly,Micrognathia,Low-set ears,Atresia of the external auditory canal,Anteverted nares,Bilateral conductive hearing impairment,Generalized hirsutism,Short stature,Hypotelorism,Delayed speech and language development,Heterochromia iridis,Global developmental delay,Ventricular septal defect,Pes planus,Deep philtrum
53	Alazami syndrome	HP:0000666,HP:0000964,HP:0001047,HP:0012429,HP:0012443,HP:0012736,HP:0030043,HP:0007687,HP:0008780,HP:0008936,HP:0002376,HP:0002650,HP:0000086,HP:0000218,HP:0	Horizontal nystagmus,Eczematoid dermatitis,Atopic dermatitis,Aplasia/Hypoplasia of the cerebral white matter,Abnormal brain morphology,Profound global developmental delay,Hip subluxation,Unilateral ptosis,Congenital bilateral hip dislocation,Axial

© 2025 Shyr C et al. *JAMA Network Open.*

		000272,HP:0000322,HP:0000400,HP:0000403,HP:0000448,HP:0000540,HP:0002020,HP:0006829,HP:0001357,HP:0001508	hypotonia,Developmental regression,Scoliosis,Ectopic kidney,High palate,Malar flattening,Short philtrum,Macrotia,Recurrent otitis media,Prominent nose,Hypermetropia,Gastroesophageal reflux,Severe muscular hypotonia,Plagiocephaly,Failure to thrive
54	CILIARY DYSKINESIA, PRIMARY, 7; CILD7	HP:0011108,HP:0012265,HP:0410018,HP:0001696	Recurrent sinusitis,Ciliary dyskinesia,Recurrent ear infections,Situs inversus totalis
55	Intellectual developmental disorder with or without epilepsy or cerebellar ataxia	HP:0007074,HP:0010754,HP:0000322,HP:0000545,HP:0001251,HP:0001263,HP:0001290,HP:0001317,HP:0001508,HP:0002376,HP:0003763,HP:0004322,HP:0000821,HP:0001250,HP:0002015	Thick corpus callosum,Abnormality of the temporomandibular joint,Short philtrum,Myopia,Ataxia,Global developmental delay,Generalized hypotonia,Abnormal cerebellum morphology,Failure to thrive,Developmental regression,Bruxism,Short stature,Hypothyroidism,Seizure,Dysphagia
56	Amyotrophic Lateral Sclerosis	HP:0000002,HP:0200136,HP:0007178,HP:0001257,HP:0002366,HP:0002380,HP:0002493	Abnormality of body height,Oral-pharyngeal dysphagia,Motor polyneuropathy,Spasticity,Abnormal lower motor neuron morphology,Fasciculations,Upper motor neuron dysfunction
57	CACNA1A-related condition	HP:0001251,HP:0004969,HP:0005162,HP:0000483,HP:0006855,HP:0007380,HP:0010863,HP:0011345,HP:0002121,HP:0002136,HP:0000565,HP:0001249,HP:0031728,HP:0045056,HP:0002427,HP:0002650	Ataxia,Peripheral pulmonary artery stenosis,Abnormal left ventricular function,Astigmatism,Cerebellar vermis atrophy,Facial telangiectasia,Receptive language delay,Moderate expressive language delay,Generalized non-motor (absence) seizure,Broad-based gait,Esotropia,Intellectual disability,Mild hypermetropia,Abnormal circulating alpha-fetoprotein concentration,Expressive aphasia,Scoliosis
58	Mental retardation, autosomal dominant 6, with or without seizures; MRD6	HP:0002650,HP:0002673,HP:0000664,HP:0000717,HP:0006380,HP:0001290,HP:0001531,HP:0100704,HP:0000020,HP:0000490,HP:0011344,H	Scoliosis,Coxa valga,Synophrys,Autism,Knee flexion contracture,Generalized hypotonia,Failure to thrive in infancy,Cerebral visual impairment,Urinary incontinence,Deeply set eye,Severe

© 2025 Shyr C et al. *JAMA Network Open.*

		P:0100543,HP:0001845,HP:0002015,HP:0002019,HP:0002020	global developmental delay,Cognitive impairment,Overlapping toe,Dysphagia,Constipation,Gastroesophageal reflux
59	3MCC Deficiency	HP:0100864,HP:0410263,HP:0500135,HP:0002637,HP:0002650,HP:0012389,HP:0012692,HP:0031717,HP:0031936,HP:0033111,HP:0007105,HP:0001250,HP:0002944,HP:0000648,HP:0000750,HP:0001249,HP:0002067	Short femoral neck,Brain imaging abnormality,Hypotryptophanemia,Cerebral ischemia,Scoliosis,Appendicular hypotonia,Focal T2 hyperintense thalamic lesion,Alternating exotropia,Delayed ability to walk,3-hydroxyisovaleric aciduria,Infantile encephalopathy,Seizure,Thoracolumbar scoliosis,Optic atrophy,Delayed speech and language development,Intellectual disability,Bradykinesia
60	Long-chain L-3 hydroxyacyl-CoA dehydrogenase deficiency (LCHAD)	HP:0001284,HP:0001531,HP:0000360,HP:0002936,HP:0003198,HP:0003201,HP:0003236,HP:0003324,HP:0003326,HP:0003390,HP:0003750,HP:0003765,HP:0002315,HP:0002566,HP:0002910,HP:0002039,HP:0002136,HP:0011471,HP:0011964,HP:0012450,HP:0031629	Areflexia,Failure to thrive in infancy,Tinnitus,Distal sensory impairment,Myopathy,Rhabdomyolysis,Elevated circulating creatine kinase concentration,Generalized muscle weakness,Myalgia,Sensory axonal neuropathy,Increased muscle fatiguability,Psoriasiform dermatitis,Headache,Intestinal malrotation,Elevated circulating hepatic transaminase concentration,Anorexia,Broad-based gait,Gastrostomy tube feeding in infancy,Intermittent painful muscle spasms,Chronic constipation,Impaired tandem gait
61	Hypophosphatasia	HP:0002942,HP:0003282,HP:0003326,HP:0001901,HP:0000939,HP:0012378,HP:0020110,HP:0025004	Thoracic kyphosis,Low alkaline phosphatase,Myalgia,Polycythemia,Osteoporosis,Fatigue,Bone fracture,Hallux rigidus
62	Nephrolithiasis/osteoporosis, hypophosphatemic, 2	HP:0002942,HP:0003282,HP:0003326,HP:0001901,HP:0000939,HP:0012378,HP:0020110,HP:0025004	Thoracic kyphosis,Low alkaline phosphatase,Myalgia,Polycythemia,Osteoporosis,Fatigue,Bone fracture,Hallux rigidus
63	UBA5-related disorder(s): Spinocerebellar ataxia & Epileptic encephalopathy early infantile, 44	HP:0002072,HP:0002078,HP:0000252,HP:0000490,HP:0000506,HP:0002355,HP:0002474,HP:0002548,HP:0001257,HP:0001263,HP:0001332,HP:0001357,HP:0001508,HP:0100559,HP:0	Chorea,Truncal ataxia,Microcephaly,Deeply set eye,Telecanthus,Difficulty walking,Expressive language delay,Parkinsonism with favorable response to dopaminergic medication,Spasticity,Global developmental delay,Dystonia,Plagiocephaly,Failure

© 2025 Shyr C et al. *JAMA Network Open.*

		012450,HP:0000565,HP:0000639,HP:0000646,HP:0000750,HP:0000964,HP:0003808,HP:0005988	to thrive,Lower limb asymmetry,Chronic constipation,Esotropia,Nystagmus,Amblyopia,Delayed speech and language development,Eczematoid dermatitis,Abnormal muscle tone,Congenital muscular torticollis
64	Wieacker-Wolff syndrome	HP:0007340,HP:0009778,HP:0011448,HP:0100874,HP:0000565,HP:0000582,HP:0000954,HP:0001249,HP:0001250,HP:0000347,HP:0000369,HP:0000414,HP:0000483,HP:0001263,HP:0001290,HP:0001344,HP:0001382,HP:0001385,HP:0012742,HP:0012746,HP:0040211,HP:0001773,HP:0002144	Lower limb muscle weakness,Short thumb,Ankle clonus,Thick hair,Esotropia,Upslanted palpebral fissure,Single transverse palmar crease,Intellectual disability,Seizure,Micrognathia,Low-set ears,Bulbous nose,Astigmatism,Global developmental delay,Generalized hypotonia,Absent speech,Joint hypermobility,Hip dysplasia,Thin fingernail,Thin toenail,Abnormal skin morphology of the palm,Short foot,Tethered cord
65	Hypermobile Ehlers-Danlos Syndrome	HP:0012332,HP:0012378,HP:0034997,HP:0003236,HP:0003323,HP:0003376,HP:0003710,HP:0009830,HP:0001278	Abnormal autonomic nervous system physiology,Fatigue,Reduced intraepidermal small nerve fiber density,Elevated circulating creatine kinase concentration,Progressive muscle weakness,Steppage gait,Exercise-induced muscle cramps,Peripheral neuropathy,Orthostatic hypotension
66	Frontotemporal dementia and/or amyotrophic lateral sclerosis 1	HP:0002174,HP:0002186,HP:0002353,HP:0002354,HP:0002359,HP:0002381,HP:0002069,HP:0002141,HP:0000713,HP:0000718,HP:0000737,HP:0000739,HP:0000751,HP:0010525,HP:0010864,HP:0100543,HP:0001266,HP:0001298	Postural tremor,Apraxia,EEG abnormality,Memory impairment,Frequent falls,Aphasia,Bilateral tonic-clonic seizure,Gait imbalance,Agitation,Aggressive behavior,Irritability,Anxiety,Personality changes,Finger agnosia,Intellectual disability, severe,Cognitive impairment,Choreoathetosis,Encephalopathy
67	Coffin-Siris syndrome 1	HP:0002198,HP:0011869,HP:0032152,HP:0001252,HP:0001263,HP:0001320,HP:0001508,HP:0001601,HP:0002079,HP:0000565,HP:0000629,HP:0000639,HP:0000680,HP:0000729,HP:0	Dilated fourth ventricle,Abnormal platelet function,Keratosis pilaris,Hypotonia,Global developmental delay,Cerebellar vermis hypoplasia,Failure to thrive,Laryngomalacia,Hypoplasia of the corpus callosum,Esotropia,Periorbital fullness,Nystagmus,Delayed eruption

© 2025 Shyr C et al. *JAMA Network Open.*

		000750,HP:0001250,HP:0007291,HP:0008689,HP:0008872,HP:0009747,HP:0010862,HP:0004322,HP:0000076,HP:0000179,HP:0000218,HP:0000219,HP:0000286,HP:0000368,HP:0000455,HP:0000486,HP:0000494,HP:0000527	of primary teeth,Autistic behavior,Delayed speech and language development,Seizure,Posterior fossa cyst,Bilateral cryptorchidism,Feeding difficulties in infancy,Lumbosacral hirsutism,Delayed fine motor development,Short stature,Vesicoureteral reflux,Thick lower lip vermilion,High palate,Thin upper lip vermilion,Epicanthus,Low-set, posteriorly rotated ears,Broad nasal tip,Strabismus,Downslanted palpebral fissures,Long eyelashes
68	Mental retardation, X-linked, with cerebellar hypoplasia and distinctive facial appearance	HP:0007018,HP:0007598,HP:0008058,HP:0010864,HP:0000577,HP:0000691,HP:0000718,HP:0000729,HP:0000957,HP:0001250,HP:0001836,HP:0001845,HP:0001848,HP:0002119,HP:0002132,HP:0001263,HP:0001319,HP:0001321,HP:0000238,HP:0000377,HP:0006349	Attention deficit hyperactivity disorder,Bilateral single transverse palmar creases,Aplasia/Hypoplasia of the optic nerve,Intellectual disability, severe,Exotropia,Microdontia,Aggressive behavior,Autistic behavior,Cafe-au-lait spot,Seizure,Camptodactyly of toe,Overlapping toe,Calcaneovalgus deformity,Ventriculomegaly,Porencephalic cyst,Global developmental delay,Neonatal hypotonia,Cerebellar hypoplasia,Hydrocephalus,Abnormal pinna morphology,Agenesis of permanent teeth
69	tubulointerstitial nephropathy	HP:0002194,HP:0001251,HP:0001288,HP:0001337,HP:0012622,HP:0100022	Delayed gross motor development,Ataxia,Gait disturbance,Tremor,Chronic kidney disease,Abnormality of movement
70	You-Hoover-Fong syndrome	HP:0011448,HP:0012098,HP:0001263,HP:0001319,HP:0001336,HP:0100704,HP:0001838,HP:0001845,HP:0001864,HP:0001998,HP:0002033,HP:0002816,HP:0000162,HP:0000218,HP:0000252,HP:0000347,HP:0000565,HP:0000960	Ankle clonus,Edema of the dorsum of feet,Global developmental delay,Neonatal hypotonia,Myoclonus,Cerebral visual impairment,Rocker bottom foot,Overlapping toe,Clinodactyly of the 5th toe,Neonatal hypoglycemia,Poor suck,Genu recurvatum,Glossoptosis,High palate,Microcephaly,Micrognathia,Esotropia,Sacral dimple
71	Autism	HP:0007598,HP:0010044,HP:0010499,HP:0011003,HP:0005101,HP:0001634,HP:0000717,HP:0000722,HP:0001187,HP:0001249,HP:0002616,HP:0002650	Bilateral single transverse palmar creases,Short 4th metacarpal,Patellar subluxation,High myopia,High-frequency hearing impairment,Mitral valve prolapse,Autism,Compulsive behaviors,Hyperextensibility of the finger joints,Intellectual

© 2025 Shyr C et al. *JAMA Network Open.*

			disability,Aortic root aneurysm,Scoliosis
72	MITOCHONDRIAL MYOPATHY, ENCEPHALOPATHY, LACTIC ACIDOSIS, AND STROKE-LIKE EPISODES; MELAS	HP:0001250,HP:0001268,HP:0003200,HP:0003737	Seizure,Mental deterioration,Ragged-red muscle fibers,Mitochondrial myopathy
73	NEURODEVELOPMENTAL DISORDER WITH HYPOTONIA, BRAIN ANOMALIES, DISTINCTIVE FACIES, AND ABSENT LANGUAGE; NEDHAFA	HP:0000592,HP:0000609,HP:0000729,HP:0000821,HP:0000824,HP:0000954,HP:0001249,HP:0410209,HP:0006610,HP:0032388,HP:0032794,HP:0002079,HP:0000218,HP:0000252,HP:0000324,HP:0000505,HP:0001263,HP:0001290,HP:0001385	Blue sclerae,Optic nerve hypoplasia,Autistic behavior,Hypothyroidism,Decreased response to growth hormone stimulation test,Single transverse palmar crease,Intellectual disability,Folate deficiency in CSF,Wide intermamillary distance,Periventricular nodular heterotopia,Myoclonic seizure,Hypoplasia of the corpus callosum,High palate,Microcephaly,Facial asymmetry,Visual impairment,Global developmental delay,Generalized hypotonia,Hip dysplasia
74	pseudohypoparathyroidism	HP:0002099,HP:0002148,HP:0003165,HP:0004322,HP:0004442,HP:0006889,HP:0007018,HP:0010862,HP:0011330,HP:0002779,HP:0002901,HP:0000243,HP:0000486,HP:0000646,HP:0000821,HP:0000843,HP:0001156	Asthma,Hypophosphatemia,Elevated circulating parathyroid hormone level,Short stature,Sagittal craniosynostosis,Intellectual disability, borderline,Attention deficit hyperactivity disorder,Delayed fine motor development,Metopic synostosis,Tracheomalacia,Hypocalcemia,Trigonocephaly,Strabismus,Amblyopia,Hypothyroidism,Hyperparathyroidism,Brachydactyly
75	Charcot-Marie-Tooth disease, axonal, type 2FF	HP:0001270,HP:0001284,HP:0001288,HP:0002515,HP:0007178,HP:0007267,HP:0008081,HP:0009073,HP:0003324,HP:0003554,HP:0033201	Motor delay,Areflexia,Gait disturbance,Waddling gait,Motor polyneuropathy,Chronic axonal neuropathy,Pes valgus,Progressive proximal muscle weakness,Generalized muscle weakness,Type 2 muscle fiber atrophy,Biceps hyporeflexia
76	HYPOTONIA, INFANTILE, WITH PSYCHOMOTOR RETARDATION AND CHARACTERISTI	HP:0002518,HP:0002020,HP:0002079,HP:0002119,HP:0004938,HP:0012378,HP:0012762,HP:0008872,HP:0010292,HP:001	Abnormal periventricular white matter morphology,Gastroesophageal reflux,Hypoplasia of the corpus callosum,Ventriculomegaly,Tortuous cerebral arteries,Fatigue,Cerebral white matter atrophy,Feeding

© 2025 Shyr C et al. *JAMA Network Open.*

	C FACIES 3; IHPRF3	0307,HP:0010804,HP:0001252,HP:0001263,HP:0001265,HP:0001344,HP:0000565,HP:0001250,HP:0000218,HP:0000280,HP:0000341,HP:0000426,HP:0000473,HP:0000508	difficulties in infancy,Absent uvula,Stridor,Tented upper lip vermilion,Hypotonia,Global developmental delay,Hyporeflexia,Absent speech,Esotropia,Seizure,High palate,Coarse facial features,Narrow forehead,Prominent nasal bridge,Torticollis,Ptosis
77	Multiple congenital anomalies-hypotonia-seizures syndrome	HP:0003785,HP:0002133,HP:0001250,HP:0001263,HP:0001344,HP:0001348,HP:0012147,HP:0012446,HP:0012762,HP:0025455,HP:0040209,HP:0100660,HP:0200134,HP:0008936	Decreased CSF homovanillic acid concentration,Status epilepticus,Seizure,Global developmental delay,Absent speech,Brisk reflexes,Reduced quantity of Von Willebrand factor,Decreased CSF 5-methyltetrahydrofolate concentration,Cerebral white matter atrophy,Decreased CSF 5-hydroxyindolacetic acid concentration,Decreased CSF biopterin level,Dyskinesia,Epileptic encephalopathy,Axial hypotonia
78	Developmental and epileptic encephalopathy 104	HP:0000733,HP:0001249,HP:0001250,HP:0002267,HP:0002307,HP:0002353,HP:0001263,HP:0001288,HP:0001332,HP:0031956,HP:0040205,HP:0040209,HP:0100025	Motor stereotypy,Intellectual disability,Seizure,Exaggerated startle response,Drooling,EEG abnormality,Global developmental delay,Gait disturbance,Dystonia,Elevated circulating aspartate aminotransferase concentration,Decreased CSF neopterin level,Decreased CSF biopterin level,Overfriendliness
79	Primary aldosteronism, seizures, and neurologic abnormalities	HP:0000565,HP:0000505,HP:0002308,HP:0002353,HP:0002360,HP:0002474,HP:0033454,HP:0033725,HP:0010863,HP:0100660,HP:0200136,HP:0002072,HP:0004322,HP:0001250,HP:0001252,HP:0001263,HP:0001332	Esotropia,Visual impairment,Chiari malformation,EEG abnormality,Sleep abnormality,Expressive language delay,Tube feeding,Thin corpus callosum,Receptive language delay,Dyskinesia,Oral-pharyngeal dysphagia,Chorea,Short stature,Seizure,Hypotonia,Global developmental delay,Dystonia
80	Congenital disorder of glycosylation, type Iw, autosomal dominant	HP:0000717,HP:0004322,HP:0430028,HP:0007018,HP:0010831,HP:0011094	Autism,Short stature,Hyperplasia of the maxilla,Attention deficit hyperactivity disorder,Impaired proprioception,Increased overbite

81	Charcot-Marie-Tooth disease, axonal, type 2s	HP:0001288,HP:0001388,HP:0001430,HP:0003307,HP:0006802,HP:0001250,HP:0001760,HP:0001773,HP:0002077,HP:0100512,HP:0007002,HP:0008936,HP:0008959,HP:0009053,HP:0010665,HP:0011203	Gait disturbance,Abnormal calf musculature morphology,Hyperlordosis,Abnormal anterior horn cell morphology,Seizure,Abnormal foot morphology,Short foot,Migraine with aura,Decreased circulating vitamin D concentration,Motor axonal neuropathy,Axial hypotonia,Distal upper limb muscle weakness,Distal lower limb muscle weakness,Bilateral coxa valga,EEG with abnormally slow frequencies
82	Senior-Loken syndrome 5	HP:0001129,HP:0003774,HP:0000479,HP:0007481	Large central visual field defect,Stage 5 chronic kidney disease,Abnormal retinal morphology,Hyperpigmented nevi
83	Alpha-1 antitrypsin deficiency (Zz)	HP:0001278,HP:0001369,HP:0001382,HP:0012668,HP:0030880,HP:0002204,HP:0000938,HP:0002105,HP:0005584,HP:0100647	Orthostatic hypotension,Arthritis,Joint hypermobility,Vasovagal syncope,Raynaud phenomenon,Pulmonary embolism,Osteopenia,Hemoptysis,Renal cell carcinoma,Graves disease
84	ANGIOPATHY, HEREDITARY, WITH NEPHROPATHY, ANEURYSMS, AND MUSCLE CRAMPS; HANAC	HP:0001278,HP:0001369,HP:0001382,HP:0012668,HP:0030880,HP:0002204,HP:0000938,HP:0002105,HP:0005584,HP:0100647	Orthostatic hypotension,Arthritis,Joint hypermobility,Vasovagal syncope,Raynaud phenomenon,Pulmonary embolism,Osteopenia,Hemoptysis,Renal cell carcinoma,Graves disease
85	BREAST CANCER susceptibility	HP:0001278,HP:0001369,HP:0001382,HP:0012668,HP:0030880,HP:0002204,HP:0000938,HP:0002105,HP:0005584,HP:0100647	Orthostatic hypotension,Arthritis,Joint hypermobility,Vasovagal syncope,Raynaud phenomenon,Pulmonary embolism,Osteopenia,Hemoptysis,Renal cell carcinoma,Graves disease
86	CHRNA3-related disease	HP:0000616,HP:0000622,HP:0002321,HP:0004926,HP:0001662,HP:0030050,HP:0000360,HP:0000518	Miosis,Blurred vision,Vertigo,Orthostatic hypotension due to autonomic dysfunction,Bradycardia,Narcolepsy,Tinnitus,Cataract
87	DENND5B related neurodevelopmental disorder,	HP:0006532,HP:0002043,HP:0002099,HP:0001263,HP:0001290,HP:0001344,HP:0000639,HP:0001249,HP:0001250,HP:000	Recurrent pneumonia,Esophageal stricture,Asthma,Global developmental delay,Generalized hypotonia,Absent speech,Nystagmus,Intellectual disability,Seizure,Myopia,Periventricul

© 2025 Shyr C et al. *JAMA Network Open.*

		0545,HP:0006970,HP:0011069,HP:0002381,HP:0002650,HP:0002808,HP:0002870	ar leukomalacia,Supernumerary tooth,Aphasia,Scoliosis,Kyphosis,Obstructive sleep apnea
88	RAUCH-STEINDL SYNDROME; RAUST	HP:0006919,HP:0007018,HP:0010529,HP:0000717,HP:0000729,HP:0002020,HP:0002099,HP:0012444,HP:0002384,HP:0002616,HP:0002808,HP:0001250,HP:0001252,HP:0001263,HP:0001508,HP:0000252,HP:0004322	Abnormal aggressive, impulsive or violent behavior,Attention deficit hyperactivity disorder,Echolalia,Autism,Autistic behavior,Gastroesophageal reflux,Asthma,Brain atrophy,Focal impaired awareness seizure,Aortic root aneurysm,Kyphosis,Seizure,Hypotonia,Global developmental delay,Failure to thrive,Microcephaly,Short stature
89	CONGENITAL DISORDER OF GLYCOSYLATION, TYPE Iy; CDG1Y	HP:0000574,HP:0001249,HP:0001250,HP:0005001,HP:0001263,HP:0001290,HP:0001488,HP:0006919,HP:0012471,HP:0000252,HP:0000347,HP:0000358,HP:0000448	Thick eyebrow,Intellectual disability,Seizure,Recurrent patellar dislocation,Global developmental delay,Generalized hypotonia,Bilateral ptosis,Abnormal aggressive, impulsive or violent behavior,Thick vermilion border,Microcephaly,Micrognathia,Posteriorly rotated ears,Prominent nose
90	Temple syndrome	HP:0001252,HP:0001263,HP:0001513,HP:0002756,HP:0002829,HP:0000311,HP:0000348,HP:0000463,HP:0002992,HP:0003701,HP:0004209,HP:0004322,HP:0012378,HP:0000678,HP:0000957,HP:0001182,HP:0001629,HP:0001773,HP:0200055,HP:0200141,HP:0009553,HP:0010465	Hypotonia,Global developmental delay,Obesity,Pathologic fracture,Arthralgia,Round face,High forehead,Anteverted nares,Abnormal tibia morphology,Proximal muscle weakness,Clinodactyly of the 5th finger,Short stature,Fatigue,Dental crowding,Cafe-au-lait spot,Tapered finger,Ventricular septal defect,Short foot,Small hand,Small, conical teeth,Abnormality of the hairline,Precocious puberty in females

eAppendix. Example Clinical Summary

"One liner": 6-year-old male with a history of global developmental delay, hypotonia, dysmorphic facial features including hypertelorism, low-set ears, and recurrent respiratory infections.

Category of Primary Condition: Neurology

Begin narrative summary of the patient's condition: Born at 38 weeks via emergency C-section due to fetal distress. Apgar scores 5 and 7 at 1 and 5 minutes. NICU stay for respiratory distress requiring CPAP for 48 hours. Noted to have hypotonia, poor suck, and feeding difficulties from birth. Early milestones significantly delayed. Rolled at 8 months, sat at 18 months, nonverbal, not walking independently. Recurrent otitis media with bilateral myringotomy tubes placed at 3 years old. Recurrent upper respiratory infections and two hospitalizations for pneumonia. Severe constipation requiring daily stool softeners. Behavioral concerns include hyperactivity, sensory-seeking behavior, and repetitive hand movements.

Family history:
-Mom: Anxiety, migraines
-Dad: Speech delay as a child
-Brother: Healthy
-Maternal Aunt: Intellectual disability, epilepsy
-Paternal GM: Autoimmune disorder

Pediatric Percentiles:
-Height (10%)
-Weight (8%)
-Head circumference (3%)

Measurements:
-Intercanthal distance: 3.4 cm (75-97%)
-Interpupillary distance: 5.2 cm (50-75%)
-Outer canthal distance: 9.5 cm (97%)
-Palpebral fissure length: 2.3 cm (10-25%)
-Right ear: 4.2 cm (<3%)
-Left ear: 4.3 cm (<3%)

Prior Genetic Testing:
-WES: No pathogenic variants identified
-Chromosomal Microarray: Normal
-Fragile X testing: Negative

Known prior evaluations:
-Brain MRI (1/2024): Mild cerebellar atrophy, delayed myelination
-Echocardiogram: Mild pulmonary valve stenosis, stable
-EEG (6/2022): Mild diffuse cerebral dysfunction without epileptiform discharges
-Ophthalmology (2021): Mild optic nerve hypoplasia, no significant visual impairment

© 2025 Shyr C et al. *JAMA Network Open.*

eTable 2. Representative Examples of the Bond Scoring Method for Large Language Model (LLM)-Generated Differential Diagnoses

**Key Features (Human Phenotype Ontology Terms)**	**Final Diagnosis**	**LLM-Generated Differential**	**Score (based on system by Bond *et al.*[3])**
Postural tremor, Apraxia, EEG abnormality, Memory impairment, Frequent falls, Aphasia, Bilateral tonic-clonic seizure, Gait imbalance, Agitation, Aggressive behavior, Irritability, Anxiety, Personality changes, Finger agnosia, Intellectual disability, severe, Cognitive impairment, Choreoathetosis, Encephalopathy	Frontotemporal Dementia and/or Amyotrophic Lateral Sclerosis	### Genetic Diagnostic Hypotheses<br><br>1. **Frontotemporal Dementia (FTD):**<br>- The patient's personality changes, cognitive decline, and imaging findings of frontal and temporal atrophy could suggest FTD. However, the lack of a clear family history makes this less likely.<br>- **Next Steps:** Genetic testing for mutations in the MAPT, GRN, and C9orf72 genes, which are associated with FTD.<br><br>2. **Amyotrophic Lateral Sclerosis (ALS):**<br>- The family history of ALS and the patient's neurological symptoms raise the possibility of familial ALS. However, the patient's presentation is atypical for ALS.<br>- **Next Steps:** Genetic testing for ALS-associated genes such as SOD1, TARDBP, FUS, and C9orf72.<br><br>### Non-Genetic Diagnostic Hypotheses<br><br>1. **Chronic Traumatic Encephalopathy (CTE):**<br>- The patient's history of concussions and cognitive and behavioral changes could suggest CTE, which is associated with repeated head trauma.<br>- **Next Steps:** Neuropsychological testing and continued monitoring of symptoms, as there is currently no definitive test for CTE.	**5 (the final diagnosis was suggested in the differential)**
Large central visual field defect, Stage 5 chronic kidney disease, Abnormal retinal morphology, Hyperpigmented nevi	Senior-Løken Syndrome 5	### Genetic Diagnostic Hypotheses:<br><br>1. **Bardet-Biedl Syndrome (BBS)**<br>- Reasoning: BBS is a ciliopathy with symptoms that can include retinal degeneration, kidney disease, and other organ anomalies. The presence of retinal degeneration and chronic kidney disease (CKD) progressing to end-stage renal disease (ESRD) aligns with the phenotypic spectrum of BBS.<br>- Next Steps: Perform targeted genetic testing for BBS genes (such as BBS1, BBS10, etc.) to identify pathogenic variants. Consider a renal biopsy to characterize the nature of the	**4 (the suggestions included something very close, but not exact)**<br><br>In this example, the model identified Senior-Løken Syndrome correctly but not the specific genetic subtype, which would

© 2025 Shyr C et al. *JAMA Network Open.*

		kidney disease, as certain histological findings can be suggestive of BBS.<br><br>2. **Senior-Løken Syndrome**<br>- Reasoning: This syndrome is a rare ciliopathy that combines a nephronophthisis (leading to CKD and ESRD) with a retinal dystrophy similar to Leber congenital amaurosis. The patient's visual impairment since birth and kidney disease are consistent with this diagnosis.<br>- Next Steps: Genetic testing for mutations in genes associated with Senior-Løken Syndrome, such as NPHP1, IQCB1, and others. Renal ultrasound may show small and echogenic kidneys, and a renal biopsy may demonstrate interstitial fibrosis and tubular atrophy.<br><br>### Non-Genetic Diagnostic Hypotheses:<br><br>1. **Chronic Interstitial Nephritis**<br>- Reasoning: Chronic interstitial nephritis can lead to CKD and ESRD. It could be secondary to an unrecognized environmental exposure or medication use. The retinal degeneration may be an unrelated finding or secondary to a shared etiology.<br>- Next Steps: A renal biopsy could help confirm the diagnosis and characterize the extent of interstitial fibrosis and tubular atrophy.	require confirmation through genetic testing.
Polyneuropathy, Muscle weakness, Myopathy, Muscle spasm, EMG: chronic denervation signs, Chronic constipation	Lambert Eaton Myasthenic Syndrome	### Genetic Diagnostic Hypotheses:<br><br>1. **Charcot-Marie-Tooth Disease Type 2A (CMT2A)**:<br>- **Reasoning**: The patient has a pathogenic heterozygous deletion in the MFN2 gene (exons 7-8), which is associated with CMT2A, an autosomal dominant disorder characterized by muscle weakness, atrophy, and sensory neuropathy. The clinical presentation of progressive muscle weakness, myopathy, and neuropathy aligns with CMT2A.<br>- **Next Steps**: Confirmatory genetic counseling and testing for other family members to assess for the presence of the MFN2 deletion and correlate with any clinical symptoms.<br>### Non-Genetic Diagnostic Hypotheses:<br><br>1. **Autoimmune Neuromuscular Disorder**:<br>- **Reasoning**: The patient's symptoms of muscle weakness, neuropathy, and autonomic dysfunction could be due to an autoimmune process. The lack of response to Mestinon does not exclude autoimmune neuromuscular	**3 (the suggestions included something closely related that might have been helpful)**<br><br>In this example, the next-step recommendations for the hypothesis, "autoimmune neuromuscular disorder," correctly suggested the underlying physiologic mechanism and recommended testing for

© 2025 Shyr C et al. *JAMA Network Open.*

		disorders, as not all patients respond to this medication.<br>- **Next Steps**: Consider testing for antibodies associated with autoimmune neuromuscular disorders, such as acetylcholine receptor antibodies, muscle-specific kinase (MuSK) antibodies, and voltage-gated calcium channel (VGCC) antibodies.	antibodies to voltage-gated calcium channels.
Lower limb muscle weakness, Short thumb, Ankle clonus, Thick hair, Esotropia, Upslanted palpebral fissure, Single transverse palmar crease, Intellectual disability, Seizure, Micrognathia, Low-set ears, Bulbous nose, Astigmatism, Global developmental delay, Generalized hypotonia, Absent speech, Joint hypermobility, Hip dysplasia, Thin fingernail, Thin toenail, Abnormal skin morphology of the palm, Short foot, Tethered cord	Wieacker-Wolff Syndrome	### Genetic Diagnostic Hypotheses:<br><br>1. **Hereditary Spastic Paraplegia (HSP)**<br>- Reasoning: The MRI findings of symmetric abnormal increased T2 signal within the posterior limbs of the internal capsules and the question of hereditary spastic paraplegia support this hypothesis. HSP is characterized by progressive weakness and spasticity of the legs, which aligns with the patient's lower leg weakness.<br>- Next Steps: Genetic testing for specific HSP-related genes, such as SPAST (SPG4), ATL1 (SPG3A), and others, could help confirm this diagnosis.<br><br>2. **Rett Syndrome Variant**<br>- Reasoning: Although MECP2 sequencing was normal, the patient's regression in vocalization, seizures, and hand stereotypies (curling) could suggest a variant of Rett syndrome, which predominantly affects girls and can have a variable presentation.<br>- Next Steps: Consider re-evaluating MECP2 and related genes such as CDKL5 and FOXG1 for mutations that may have been missed or new mutations that have been characterized since the last testing.<br><br>### Non-Genetic Diagnostic Hypotheses:<br><br>1. **Cerebral Palsy**<br>- Reasoning: The early hypotonia, latching difficulties, and delayed motor milestones could be indicative of cerebral palsy, a group of disorders affecting movement and muscle tone or posture caused by damage to the immature, developing brain, often before birth.<br>- Next Steps: A detailed neurological examination and review of the brain MRI findings by a pediatric neurologist to assess for signs consistent with cerebral palsy.	**2 (the suggestions included something related, but unlikely to be helpful)**<br><br>In this example, suggestions such as hereditary spastic paraplegia and Rett syndrome share overlap with Wieacker-Wolff syndrome in terms of neurological symptoms, but these suggestions are unlikely to be helpful.
Orthostatic hypotension, Arthritis, Joint hypermobility, Vasovagal	Breast cancer susceptibility due to	### Genetic Diagnostic Hypotheses:<br><br>1. **Hereditary Hemorrhagic Telangiectasia (HHT)**	**0 (no suggestions were close to the final diagnosis)**

© 2025 Shyr C et al. *JAMA Network Open.*

syncope, Raynaud phenomenon, Pulmonary embolism, Osteopenia, Hemoptysis, Renal cell carcinoma, Graves disease	mutation in *BARD1*	- Reasoning: The recurrent episodes of hemoptysis and history of severe bleeding, including a life-threatening hemorrhage post-childbirth, could be indicative of HHT, which is associated with arteriovenous malformations and bleeding. The presence of pulmonary arterial hypertension (PAH) and abnormal vascular findings on imaging could also be consistent with this diagnosis.<br>- Next Steps: Genetic testing for mutations in genes commonly associated with HHT, such as ENG, ACVRL1, and SMAD4, should be considered to confirm the diagnosis.<br><br>### Non-Genetic Diagnostic Hypotheses:<br><br>1. **Chronic Thromboembolic Pulmonary Hypertension (CTEPH)**<br>- Reasoning: The history of bilateral pulmonary emboli and enlargement of the main pulmonary artery could indicate CTEPH, a form of pulmonary hypertension caused by chronic blood clots in the lungs.<br>- Next Steps: A V/Q scan and pulmonary angiography could be considered to assess for CTEPH, along with consultation with a pulmonologist specializing in pulmonary hypertension.	

© 2025 Shyr C et al. *JAMA Network Open.*
