# Supplementary material for: Large Language Models for Rare Disease Diagnosis at the Undiagnosed Diseases Network
Source: JAMA Netw Open. 2025 Aug 22;8(8):e2528538. doi: 10.1001/jamanetworkopen.2025.28538 (PMC12374213; doi:10.1001/jamanetworkopen.2025.28538)
Supplement: Supplement 2. — Nonauthor Collaborators. Undiagnosed Diseases Network Members [file jamanetwopen-e2528538-s002.pdf]

Supplemental Online Content: Nonauthor Collaborators
*First name, last name, and suffix (if applicable) are required and will appear in PubMed.

*Group Name(s): Undiagnosed Diseases Network							
***First Name and Middle Initial(s)**	***Last Name**	***Suffix (eg, Jr, III)**	Academic Degrees	Institution	Location (city, state/province, country)	Role or Contribution, eg, chair, principal investigator	Group (if more than 1 Group listed in the byline) and/or Subgroup (eg, Steering Committee)
Alyssa A.	Tran						Undiagnosed Diseases Network
Arjun	Tarakad						Undiagnosed Diseases Network
Ashok	Balasubramanyam						Undiagnosed Diseases Network
Brendan H.	Lee						Undiagnosed Diseases Network
Carlos A.	Bacino						Undiagnosed Diseases Network
Daryl A.	Scott						Undiagnosed Diseases Network
Elaine	Seto						Undiagnosed Diseases Network
Gary D.	Clark						Undiagnosed Diseases Network
Hongzheng	Dai						Undiagnosed Diseases Network
Hsiao-Tuan	Chao						Undiagnosed Diseases Network
Ivan	Chinn						Undiagnosed Diseases Network
James P.	Orengo						Undiagnosed Diseases Network
Jennifer E.	Posey						Undiagnosed Diseases Network
Jill A.	Rosenfeld						Undiagnosed Diseases Network
Kim	Worley						Undiagnosed Diseases Network

*First name, last name, and suffix (if applicable) are required and will appear in PubMed.

***First Name and Middle Initial(s)**	***Last Name**	***Suffix (eg, Jr, III)**	Academic Degrees	Institution	Location (city, state/province, country)	Role or Contribution, eg, chair, principal investigator	Group (if more than 1 Group listed in the byline) and/or Subgroup (eg, Steering Committee)
Lindsay C.	Burrage						Undiagnosed Diseases Network
Lisa T.	Emrick						Undiagnosed Diseases Network
Lorraine	Potocki						Undiagnosed Diseases Network
Monika Weisz	Hubshman						Undiagnosed Diseases Network
Richard A.	Lewis						Undiagnosed Diseases Network
Ronit	Marom						Undiagnosed Diseases Network
Seema R.	Lalani						Undiagnosed Diseases Network
Shamika	Ketkar						Undiagnosed Diseases Network
Tiphanie P.	Vogel						Undiagnosed Diseases Network
William J.	Craigen						Undiagnosed Diseases Network
Jared	Sninsky						Undiagnosed Diseases Network
Lauren	Blieden						Undiagnosed Diseases Network
Sandesh	Nagamani						Undiagnosed Diseases Network
Hugo J.	Bellen						Undiagnosed Diseases Network
Michael F.	Wangler						Undiagnosed Diseases Network
Oguz	Kanca						Undiagnosed Diseases Network

Supplemental Online Content: Nonauthor Collaborators
*First name, last name, and suffix (if applicable) are required and will appear in PubMed.

***First Name and Middle Initial(s)**	***Last Name**	***Suffix (eg, Jr, III)**	Academic Degrees	Institution	Location (city, state/province, country)	Role or Contribution, eg, chair, principal investigator	Group (if more than 1 Group listed in the byline) and/or Subgroup (eg, Steering Committee)
Shinya	Yamamoto						Undiagnosed Diseases Network
Christine M.	Eng						Undiagnosed Diseases Network
Patricia A.	Ward						Undiagnosed Diseases Network
Pengfei	Liu						Undiagnosed Diseases Network
Adeline	Vanderver						Undiagnosed Diseases Network
Cara	Skraban						Undiagnosed Diseases Network
Edward	Behrens						Undiagnosed Diseases Network
Gonench	Kilich						Undiagnosed Diseases Network
Kathleen	Sullivan						Undiagnosed Diseases Network
Kelly	Hassey						Undiagnosed Diseases Network
Ramakrishnan	Rajagopalan						Undiagnosed Diseases Network
Rebecca	Ganetzky						Undiagnosed Diseases Network
Vishnu	Cuddapah						Undiagnosed Diseases Network
Anna	Raper						Undiagnosed Diseases Network
Daniel J.	Rader						Undiagnosed Diseases Network
Giorgio	Sirugo						Undiagnosed Diseases Network

*First name, last name, and suffix (if applicable) are required and will appear in PubMed.

*First Name and Middle Initial(s)	*Last Name	*Suffix (eg, Jr, III)	Academic Degrees	Institution	Location (city, state/province, country)	Role or Contribution, eg, chair, principal investigator	Group (if more than 1 Group listed in the byline) and/or Subgroup (eg, Steering Committee)
Vaidehi	Jobanputra						Undiagnosed Diseases Network
Allyn	McConkie-Rosell						Undiagnosed Diseases Network
Kelly	Schoch						Undiagnosed Diseases Network
Mohamad	Mikati						Undiagnosed Diseases Network
Nicole M.	Walley						Undiagnosed Diseases Network
Rebecca C.	Spillmann						Undiagnosed Diseases Network
Vandana	Shashi						Undiagnosed Diseases Network
Alan H.	Beggs						Undiagnosed Diseases Network
Calum A.	MacRae						Undiagnosed Diseases Network
David A.	Sweetser						Undiagnosed Diseases Network
Deepak A.	Rao						Undiagnosed Diseases Network
Edwin K.	Silverman						Undiagnosed Diseases Network
Elizabeth L.	Fieg						Undiagnosed Diseases Network
Frances	High						Undiagnosed Diseases Network
Gerard T.	Berry						Undiagnosed Diseases Network
Ingrid A.	Holm						Undiagnosed Diseases Network

Supplemental Online Content: Nonauthor Collaborators
*First name, last name, and suffix (if applicable) are required and will appear in PubMed.

***First Name and Middle Initial(s)**	***Last Name**	***Suffix (eg, Jr, III)**	Academic Degrees	Institution	Location (city, state/province, country)	Role or Contribution, eg, chair, principal investigator	Group (if more than 1 Group listed in the byline) and/or Subgroup (eg, Steering Committee)
J. Carl	Pallais						Undiagnosed Diseases Network
Joan M.	Stoler						Undiagnosed Diseases Network
Joseph	Loscalzo						Undiagnosed Diseases Network
Lance H.	Rodan						Undiagnosed Diseases Network
Laurel A.	Cobban						Undiagnosed Diseases Network
Lauren C.	Briere						Undiagnosed Diseases Network
Matthew	Coggins						Undiagnosed Diseases Network
Melissa	Walker						Undiagnosed Diseases Network
Richard L.	Maas						Undiagnosed Diseases Network
Susan	Korrick						Undiagnosed Diseases Network
Jessica	Douglas						Undiagnosed Diseases Network
Cecilia	Esteves						Undiagnosed Diseases Network
Emily	Glanton						Undiagnosed Diseases Network
Isaac S.	Kohane						Undiagnosed Diseases Network
Kimberly	LeBlanc						Undiagnosed Diseases Network
Rachel	Mahoney						Undiagnosed Diseases Network

Supplemental Online Content: Nonauthor Collaborators
*First name, last name, and suffix (if applicable) are required and will appear in PubMed.

***First Name and Middle Initial(s)**	***Last Name**	***Suffix (eg, Jr, III)**	Academic Degrees	Institution	Location (city, state/province, country)	Role or Contribution, eg, chair, principal investigator	Group (if more than 1 Group listed in the byline) and/or Subgroup (eg, Steering Committee)
Shamil R.	Sunyaev						Undiagnosed Diseases Network
Shilpa N.	Kobren						Undiagnosed Diseases Network
Brett H.	Graham						Undiagnosed Diseases Network
Erin	Conboy						Undiagnosed Diseases Network
Francesco	Vetrini						Undiagnosed Diseases Network
Kayla M.	Treat						Undiagnosed Diseases Network
Khurram	Liaqat						Undiagnosed Diseases Network
Lili	Mantcheva						Undiagnosed Diseases Network
Stephanie M.	Ware						Undiagnosed Diseases Network
Breanna	Mitchell						Undiagnosed Diseases Network
Brendan C.	Lanpher						Undiagnosed Diseases Network
Devin	Oglesbee						Undiagnosed Diseases Network
Eric	Klee						Undiagnosed Diseases Network
Filippo Pinto e	Vairo						Undiagnosed Diseases Network
Ian R.	Lanza						Undiagnosed Diseases Network
Kahlen	Darr						Undiagnosed Diseases Network

*First name, last name, and suffix (if applicable) are required and will appear in PubMed.

***First Name and Middle Initial(s)**	***Last Name**	***Suffix (eg, Jr, III)**	Academic Degrees	Institution	Location (city, state/province, country)	Role or Contribution, eg, chair, principal investigator	Group (if more than 1 Group listed in the byline) and/or Subgroup (eg, Steering Committee)
Lindsay	Mulvihill						Undiagnosed Diseases Network
Lisa	Schimmenti						Undiagnosed Diseases Network
Queenie	Tan						Undiagnosed Diseases Network
Surendra	Dasari						Undiagnosed Diseases Network
Abdul	Elkadri						Undiagnosed Diseases Network
Brett	Bordini						Undiagnosed Diseases Network
Donald	Basel						Undiagnosed Diseases Network
James	Verbsky						Undiagnosed Diseases Network
Julie	McCarrier						Undiagnosed Diseases Network
Michael	Muriello						Undiagnosed Diseases Network
Michael	Zimmermann						Undiagnosed Diseases Network
Adriana	Rebelo						Undiagnosed Diseases Network
Carson A.	Smith						Undiagnosed Diseases Network
Deborah	Barbouth						Undiagnosed Diseases Network
Guney	Bademci						Undiagnosed Diseases Network
Joanna M.	Gonzalez						Undiagnosed Diseases Network

Supplemental Online Content: Nonauthor Collaborators
*First name, last name, and suffix (if applicable) are required and will appear in PubMed.

*First Name and Middle Initial(s)	*Last Name	*Suffix (eg, Jr, III)	Academic Degrees	Institution	Location (city, state/province, country)	Role or Contribution, eg, chair, principal investigator	Group (if more than 1 Group listed in the byline) and/or Subgroup (eg, Steering Committee)
Kumarie	Latchman						Undiagnosed Diseases Network
LéShon	Peart						Undiagnosed Diseases Network
Mustafa	Tekin						Undiagnosed Diseases Network
Nicholas	Borja						Undiagnosed Diseases Network
Stephan	Zuchner						Undiagnosed Diseases Network
Stephanie	Bivona						Undiagnosed Diseases Network
Willa	Thorson						Undiagnosed Diseases Network
Herman	Taylor						Undiagnosed Diseases Network
Rakale C.	Quarells						Undiagnosed Diseases Network
Ayuko	Iverson						Undiagnosed Diseases Network
Bruce	Gelb						Undiagnosed Diseases Network
Charlotte	Cunningham-Rundles						Undiagnosed Diseases Network
Eric	Gayle						Undiagnosed Diseases Network
Joanna	Jen						Undiagnosed Diseases Network
Louise	Bier						Undiagnosed Diseases Network
Mafalda	Barbosa						Undiagnosed Diseases Network

Supplemental Online Content: Nonauthor Collaborators
*First name, last name, and suffix (if applicable) are required and will appear in PubMed.

***First Name and Middle Initial(s)**	***Last Name**	***Suffix (eg, Jr, III)**	Academic Degrees	Institution	Location (city, state/province, country)	Role or Contribution, eg, chair, principal investigator	Group (if more than 1 Group listed in the byline) and/or Subgroup (eg, Steering Committee)
Manisha	Balwani						Undiagnosed Diseases Network
Mariya	Shadrina						Undiagnosed Diseases Network
Rachel	Evard						Undiagnosed Diseases Network
Saskia	Shuman						Undiagnosed Diseases Network
Susan	Shin						Undiagnosed Diseases Network
Andrea	Gropman						Undiagnosed Diseases Network
Barbara N.	Swerdzewski						Undiagnosed Diseases Network
Camilo	Toro						Undiagnosed Diseases Network
Colleen E.	Wahl						Undiagnosed Diseases Network
Donna	Novacic						Undiagnosed Diseases Network
Ellen F.	Macnamara						Undiagnosed Diseases Network
John J.	Mulvihill						Undiagnosed Diseases Network
Maria T.	Acosta						Undiagnosed Diseases Network
Precilla	D'Souza						Undiagnosed Diseases Network
Valerie V.	Maduro						Undiagnosed Diseases Network
Ben	Afzali						Undiagnosed Diseases Network

Supplemental Online Content: Nonauthor Collaborators
*First name, last name, and suffix (if applicable) are required and will appear in PubMed.

*First Name and Middle Initial(s)	*Last Name	*Suffix (eg, Jr, III)	Academic Degrees	Institution	Location (city, state/province, country)	Role or Contribution, eg, chair, principal investigator	Group (if more than 1 Group listed in the byline) and/or Subgroup (eg, Steering Committee)
Ben	Solomon						Undiagnosed Diseases Network
Cynthia J.	Tifft						Undiagnosed Diseases Network
David R.	Adams						Undiagnosed Diseases Network
Elizabeth A.	Burke						Undiagnosed Diseases Network
Francis	Rossignol						Undiagnosed Diseases Network
Heidi	Wood						Undiagnosed Diseases Network
Jiayu	Fu						Undiagnosed Diseases Network
Joie	Davis						Undiagnosed Diseases Network
Leoyklang	Petcharet						Undiagnosed Diseases Network
Lynne A.	Wolfe						Undiagnosed Diseases Network
Margaret	Delgado						Undiagnosed Diseases Network
Marie	Morimoto						Undiagnosed Diseases Network
Marla	Sabaii						Undiagnosed Diseases Network
MayChristine V.	Malicdan						Undiagnosed Diseases Network
Neil	Hanchard						Undiagnosed Diseases Network
Orpa	Jean-Marie						Undiagnosed Diseases Network

Supplemental Online Content: Nonauthor Collaborators
*First name, last name, and suffix (if applicable) are required and will appear in PubMed.

***First Name and Middle Initial(s)**	***Last Name**	***Suffix (eg, Jr, III)**	Academic Degrees	Institution	Location (city, state/province, country)	Role or Contribution, eg, chair, principal investigator	Group (if more than 1 Group listed in the byline) and/or Subgroup (eg, Steering Committee)
Wendy	Introne						Undiagnosed Diseases Network
William A.	Gahl						Undiagnosed Diseases Network
Yan	Huang						Undiagnosed Diseases Network
Andrew	Stergachis						Undiagnosed Diseases Network
Danny	Miller						Undiagnosed Diseases Network
Elisabeth	Rosenthal						Undiagnosed Diseases Network
Elizabeth	Blue						Undiagnosed Diseases Network
Elsa	Balton						Undiagnosed Diseases Network
Emily	Shelkowitz						Undiagnosed Diseases Network
Eric	Allenspach						Undiagnosed Diseases Network
Fuki M.	Hisama						Undiagnosed Diseases Network
Gail P.	Jarvik						Undiagnosed Diseases Network
Ghayda	Mirzaa						Undiagnosed Diseases Network
Ian	Glass						Undiagnosed Diseases Network
Kathleen A.	Leppig						Undiagnosed Diseases Network
Katrina	Dipple						Undiagnosed Diseases Network

Supplemental Online Content: Nonauthor Collaborators
*First name, last name, and suffix (if applicable) are required and will appear in PubMed.

***First Name and Middle Initial(s)**	***Last Name**	***Suffix (eg, Jr, III)**	Academic Degrees	Institution	Location (city, state/province, country)	Role or Contribution, eg, chair, principal investigator	Group (if more than 1 Group listed in the byline) and/or Subgroup (eg, Steering Committee)
Mark	Wener						Undiagnosed Diseases Network
Martha	Horike-Pyne						Undiagnosed Diseases Network
Michael	Bamshad						Undiagnosed Diseases Network
Peter	Byers						Undiagnosed Diseases Network
Runjun	Kumar						Undiagnosed Diseases Network
Seth	Perlman						Undiagnosed Diseases Network
Sirisak	Chanprasert						Undiagnosed Diseases Network
Virginia	Sybert						Undiagnosed Diseases Network
Wendy	Raskind						Undiagnosed Diseases Network
Nitsuh K.	Dargie						Undiagnosed Diseases Network
Chun-Hung	Chan						Undiagnosed Diseases Network
Francisco Bustos	Velasquez						Undiagnosed Diseases Network
Isum	Ward						Undiagnosed Diseases Network
Jason	Schend						Undiagnosed Diseases Network
Jennifer	Morgan						Undiagnosed Diseases Network
Megan	Bell						Undiagnosed Diseases Network

Supplemental Online Content: Nonauthor Collaborators
*First name, last name, and suffix (if applicable) are required and will appear in PubMed.

***First Name and Middle Initial(s)**	***Last Name**	***Suffix (eg, Jr, III)**	Academic Degrees	Institution	Location (city, state/province, country)	Role or Contribution, eg, chair, principal investigator	Group (if more than 1 Group listed in the byline) and/or Subgroup (eg, Steering Committee)
Miranda	Leitheiser						Undiagnosed Diseases Network
Mohamad	Saifeddine						Undiagnosed Diseases Network
Paul	Berger						Undiagnosed Diseases Network
Rachel	Li						Undiagnosed Diseases Network
Taylor	Beagle						Undiagnosed Diseases Network
Alexander	Miller						Undiagnosed Diseases Network
Beatriz	Anguiano						Undiagnosed Diseases Network
Beth A.	Martin						Undiagnosed Diseases Network
Brianna	Tucker						Undiagnosed Diseases Network
Chloe M.	Reuter						Undiagnosed Diseases Network
Devon	Bonner						Undiagnosed Diseases Network
Elijah	Kravets						Undiagnosed Diseases Network
Hector Rodrigo	Mendez						Undiagnosed Diseases Network
Holly K.	Tabor						Undiagnosed Diseases Network
Jacinda B.	Sampson						Undiagnosed Diseases Network
Jason	Hom						Undiagnosed Diseases Network

Supplemental Online Content: Nonauthor Collaborators
*First name, last name, and suffix (if applicable) are required and will appear in PubMed.

*First Name and Middle Initial(s)	*Last Name	*Suffix (eg, Jr, III)	Academic Degrees	Institution	Location (city, state/province, country)	Role or Contribution, eg, chair, principal investigator	Group (if more than 1 Group listed in the byline) and/or Subgroup (eg, Steering Committee)
Jennefer N.	Kohler						Undiagnosed Diseases Network
Jennifer	Schymick						Undiagnosed Diseases Network
John E.	Gorzynski						Undiagnosed Diseases Network
Jonathan A.	Bernstein						Undiagnosed Diseases Network
Kevin S.	Smith						Undiagnosed Diseases Network
Laura	Keehan						Undiagnosed Diseases Network
Laurens	Wiel						Undiagnosed Diseases Network
Matthew T.	Wheeler						Undiagnosed Diseases Network
Meghan C.	Halley						Undiagnosed Diseases Network
Mia	Levanto						Undiagnosed Diseases Network
Page C.	Goddard						Undiagnosed Diseases Network
Paul G.	Fisher						Undiagnosed Diseases Network
Rachel A.	Ungar						Undiagnosed Diseases Network
Raquel L.	Alvarez						Undiagnosed Diseases Network
Sara	Emami						Undiagnosed Diseases Network
Shruti	Marwaha						Undiagnosed Diseases Network

Supplemental Online Content: Nonauthor Collaborators
*First name, last name, and suffix (if applicable) are required and will appear in PubMed.

***First Name and Middle Initial(s)**	***Last Name**	***Suffix (eg, Jr, III)**	Academic Degrees	Institution	Location (city, state/province, country)	Role or Contribution, eg, chair, principal investigator	Group (if more than 1 Group listed in the byline) and/or Subgroup (eg, Steering Committee)
Stephen B	Montgomery						Undiagnosed Diseases Network
Suha	Bachir						Undiagnosed Diseases Network
Tanner D	Jensen						Undiagnosed Diseases Network
Taylor	Maurer						Undiagnosed Diseases Network
Terra R.	Coakley						Undiagnosed Diseases Network
Euan A.	Ashley						Undiagnosed Diseases Network
Ali	Al-Beshri						Undiagnosed Diseases Network
Anna	Hurst						Undiagnosed Diseases Network
Brandon M	Wilk						Undiagnosed Diseases Network
Bruce	Korf						Undiagnosed Diseases Network
Elizabeth A	Worthey						Undiagnosed Diseases Network
Kaitlin	Callaway						Undiagnosed Diseases Network
Martin	Rodriguez						Undiagnosed Diseases Network
Tammi	Skelton						Undiagnosed Diseases Network
Tarun KK	Mamidi						Undiagnosed Diseases Network
Andrew B.	Crouse						Undiagnosed Diseases Network

*First name, last name, and suffix (if applicable) are required and will appear in PubMed.

***First Name and Middle Initial(s)**	***Last Name**	***Suffix (eg, Jr, III)**	Academic Degrees	Institution	Location (city, state/province, country)	Role or Contribution, eg, chair, principal investigator	Group (if more than 1 Group listed in the byline) and/or Subgroup (eg, Steering Committee)
Jordan	Whitlock						Undiagnosed Diseases Network
Mariko	Nakano-Okuno						Undiagnosed Diseases Network
Matthew	Might						Undiagnosed Diseases Network
William E.	Byrd						Undiagnosed Diseases Network
Albert R.	La Spada						Undiagnosed Diseases Network
Changrui	Xiao						Undiagnosed Diseases Network
Elizabeth C.	Chao						Undiagnosed Diseases Network
Eric	Vilain						Undiagnosed Diseases Network
Jose	Abdenur						Undiagnosed Diseases Network
Kirsten	Blanco						Undiagnosed Diseases Network
Maija-Rikka	Steenari						Undiagnosed Diseases Network
Rebekah	Barrick						Undiagnosed Diseases Network
Richard	Chang						Undiagnosed Diseases Network
Sanaz	Attaripour						Undiagnosed Diseases Network
Suzanne	Sandmeyer						Undiagnosed Diseases Network
Tahseen	Mozaffar						Undiagnosed Diseases Network

Supplemental Online Content: Nonauthor Collaborators
*First name, last name, and suffix (if applicable) are required and will appear in PubMed.

*First Name and Middle Initial(s)	*Last Name	*Suffix (eg, Jr, III)	Academic Degrees	Institution	Location (city, state/province, country)	Role or Contribution, eg, chair, principal investigator	Group (if more than 1 Group listed in the byline) and/or Subgroup (eg, Steering Committee)
Alden	Huang						Undiagnosed Diseases Network
Andres	Vargas						Undiagnosed Diseases Network
Bianca E.	Russell						Undiagnosed Diseases Network
Brent L.	Fogel						Undiagnosed Diseases Network
Esteban C.	Dell'Angelica						Undiagnosed Diseases Network
George	Carvalho						Undiagnosed Diseases Network
Julian A.	Martínez-Agosto						Undiagnosed Diseases Network
Layal F. Abi	Farraj						Undiagnosed Diseases Network
Manish J.	Butte						Undiagnosed Diseases Network
Martin G.	Martin						Undiagnosed Diseases Network
Naghmeh	Dorrani						Undiagnosed Diseases Network
Neil H.	Parker						Undiagnosed Diseases Network
Rosario I.	Corona						Undiagnosed Diseases Network
Stanley F.	Nelson						Undiagnosed Diseases Network
Yigit	Karasozen						Undiagnosed Diseases Network
Aaron	Quinlan						Undiagnosed Diseases Network

Supplemental Online Content: Nonauthor Collaborators
*First name, last name, and suffix (if applicable) are required and will appear in PubMed.

***First Name and Middle Initial(s)**	***Last Name**	***Suffix (eg, Jr, III)**	Academic Degrees	Institution	Location (city, state/province, country)	Role or Contribution, eg, chair, principal investigator	Group (if more than 1 Group listed in the byline) and/or Subgroup (eg, Steering Committee)
Alistair	Ward						Undiagnosed Diseases Network
Ashley	Andrews						Undiagnosed Diseases Network
Corrine K.	Welt						Undiagnosed Diseases Network
Dave	Viskochil						Undiagnosed Diseases Network
Erin E.	Baldwin						Undiagnosed Diseases Network
John	Carey						Undiagnosed Diseases Network
Justin	Alvey						Undiagnosed Diseases Network
Laura	Pace						Undiagnosed Diseases Network
Lorenzo	Botto						Undiagnosed Diseases Network
Nicola	Longo						Undiagnosed Diseases Network
Paolo	Moretti						Undiagnosed Diseases Network
Rebecca	Overbury						Undiagnosed Diseases Network
Russell	Butterfield						Undiagnosed Diseases Network
Steven	Boyden						Undiagnosed Diseases Network
Thomas J.	Nicholas						Undiagnosed Diseases Network
Matt	Velinder						Undiagnosed Diseases Network

*First name, last name, and suffix (if applicable) are required and will appear in PubMed.

***First Name and Middle Initial(s)**	***Last Name**	***Suffix (eg, Jr, III)**	Academic Degrees	Institution	Location (city, state/province, country)	Role or Contribution, eg, chair, principal investigator	Group (if more than 1 Group listed in the byline) and/or Subgroup (eg, Steering Committee)
Gabor	Marth						Undiagnosed Diseases Network
Pinar	Bayrak-Toydemir						Undiagnosed Diseases Network
Rong	Mao						Undiagnosed Diseases Network
Monte	Westerfield						Undiagnosed Diseases Network
Alyson	Krokosky						Undiagnosed Diseases Network
John A.	Phillips	III					Undiagnosed Diseases Network
Kimberly	Ezell						Undiagnosed Diseases Network
Lynette	Rives						Undiagnosed Diseases Network
Joy D.	Cogan						Undiagnosed Diseases Network
Alex	Paul						Undiagnosed Diseases Network
Dana	Kiley						Undiagnosed Diseases Network
Daniel	Wegner						Undiagnosed Diseases Network
Erin	McRoy						Undiagnosed Diseases Network
Jennifer	Wambach						Undiagnosed Diseases Network
Kathy	Sisco						Undiagnosed Diseases Network
Patricia	Dickson						Undiagnosed Diseases Network

*First name, last name, and suffix (if applicable) are required and will appear in PubMed.

***First Name and Middle Initial(s)**	***Last Name**	***Suffix (eg, Jr, III)**	Academic Degrees	Institution	Location (city, state/province, country)	Role or Contribution, eg, chair, principal investigator	Group (if more than 1 Group listed in the byline) and/or Subgroup (eg, Steering Committee)
F. Sessions	Cole						Undiagnosed Diseases Network
Dustin	Baldridge						Undiagnosed Diseases Network
Jimann	Shin						Undiagnosed Diseases Network
Lilianna	Solnica-Krezel						Undiagnosed Diseases Network
Stephen C.	Pak						Undiagnosed Diseases Network
Timothy	Schedl						Undiagnosed Diseases Network
Allen	Bale						Undiagnosed Diseases Network
Carol	Oladele						Undiagnosed Diseases Network
Caroline	Hendry						Undiagnosed Diseases Network
Emily	Wang						Undiagnosed Diseases Network
Hui	Zhang						Undiagnosed Diseases Network
Lauren	Jeffries						Undiagnosed Diseases Network
María José Ortuño	Romero						Undiagnosed Diseases Network
Mark	Gerstein						Undiagnosed Diseases Network
Michele	Spencer-Manzon						Undiagnosed Diseases Network
Monkol	Lek						Undiagnosed Diseases Network

Supplemental Online Content: Nonauthor Collaborators
*First name, last name, and suffix (if applicable) are required and will appear in PubMed.

***First Name and Middle Initial(s)**	***Last Name**	***Suffix (eg, Jr, III)**	Academic Degrees	Institution	Location (city, state/province, country)	Role or Contribution, eg, chair, principal investigator	Group (if more than 1 Group listed in the byline) and/or Subgroup (eg, Steering Committee)
Nada	Derar						Undiagnosed Diseases Network
Odelya	Kaufman						Undiagnosed Diseases Network
Shrikant	Mane						Undiagnosed Diseases Network
Teodoro Jerves	Serrano						Undiagnosed Diseases Network
Vasilis	Vasiliou						Undiagnosed Diseases Network
Winston	Halstead						Undiagnosed Diseases Network
Yong-Hui	Jiang						Undiagnosed Diseases Network
