# Supplementary material for: Large Language Models for Rare Disease Diagnosis at the Undiagnosed Diseases Network
Source: JAMA Netw Open. 2025 Aug 22;8(8):e2528538. doi: 10.1001/jamanetworkopen.2025.28538 (PMC12374213; doi:10.1001/jamanetworkopen.2025.28538)
Supplement: Supplement 3. — Data Sharing Statement [file jamanetwopen-e2528538-s003.pdf]

# Data Sharing Statement

Shyr. Large Language Models for Rare Disease Diagnosis at the Undiagnosed Diseases Network. *JAMA Netw Open*. Published August 22, 2025.
doi:10.1001/jamanetworkopen.2025.28538

**Data**
**Data available:** Yes
**Data types:** Deidentified participant data
**How to access data:** Data used in this study are from the U.S. National Institutes of Health's Undiagnosed Diseases Network and contain sensitive patient information. De-identified patient data, including phenotypic and genomic data, are deposited in the database of Genotypes and Phenotypes (dbGaP) maintained by the National Institutes of Health (https://www.ncbi.nlm.nih.gov/gap/). To explore data available in the latest release, visit the UDN study page in dbGaP (https://www.ncbi.nlm.nih.gov/projects/gap/cgi-bin/study.cgi?study_id=phs001232.v7.p3). Individuals interested in accessing UDN data through dbGaP should submit a data access request. Detailed instructions for this process can be found on the NIH Scientific Data Sharing website: How to Request and Access Datasets from dbGaP (https://sharing.nih.gov/accessing-data/accessing-genomic-data/how-to-request-and-access-datasets-from-dbgap).
**When available:** With publication

**Supporting Documents**
**Document types:** None

**Additional Information**
**Who can access the data:** This data will be made available to researchers who have approval for accessing the Undiagnosed Diseases Network data through dbGaP (https://www.ncbi.nlm.nih.gov/projects/gap/cgi-bin/study.cgi?study_id=phs001232.v7.p3). For more information on how to request and access datasets from dbGaP, please visit https://sharing.nih.gov/accessing-data/accessing-genomic-data/how-to-request-and-access-datasets-from-dbgap.
**Types of analyses:** Analyses may include, but are not limited to, genetic, phenotypic, and computational studies that align with the Undiagnosed Diseases Network's mission to foster collaborative research and improve patient care.
**Mechanisms of data availability:** The data will be made available after approval of a dbGaP data access request at https://www.ncbi.nlm.nih.gov/projects/gap/cgi-bin/study.cgi?study_id=phs001232.v7.p3.
